# Worldwide Spread of Dengue Virus Type 1

**DOI:** 10.1371/journal.pone.0062649

**Published:** 2013-05-13

**Authors:** Christian Julián Villabona-Arenas, Paolo Marinho de Andrade Zanotto

**Affiliations:** Laboratory of Molecular Evolution and Bioinformatics, Department of Microbiology, Biomedical Sciences Institute, University of São Paulo, São Paulo, Brazil; University of Texas Medical Branch, United States of America

## Abstract

**Background:**

DENV-1 is one of the four viral serotypes that causes Dengue, the most common mosquito-borne viral disease of humans. The prevalence of these viruses has grown in recent decades and is now present in more than 100 countries. Limited studies document the spread of DENV-1 over the world despite its importance for human health.

**Methodology/Principal Findings:**

We used representative DENV-1 envelope gene sequences to unravel the dynamics of viral diffusion under a Bayesian phylogeographic approach. Data included strains from 45 distinct geographic locations isolated from 1944 to 2009. The estimated mean rate of nucleotide substitution was 6.56×10^−4^ substitutions/site/year. The larger genotypes (I, IV and V) had a distinctive phylogenetic structure and since 1990 they experienced effective population size oscillations. Thailand and Indonesia represented the main sources of strains for neighboring countries. Besides, Asia broadcast lineages into the Americas and the Pacific region that diverged in isolation. Also, a transmission network analysis revealed the pivotal role of Indochina in the global diffusion of DENV-1 and of the Caribbean in the diffusion over the Americas.

**Conclusions/Significance:**

The study summarizes the spatiotemporal DENV-1 worldwide spread that may help disease control.

## Introduction

Dengue virus type 1 (DENV-1) is one of the four serotypes of the arthropod borne viruses (arbovirus) that causes Dengue [1], [2]. Mosquitoes (mainly of the *Aedes* genus) transmit the virus and also infect non-human primates in a sylvatic cycle [3]–[5]. This RNA virus causes a fever that may be temporarily incapacitating and characterized by extreme pain and stiffness of the joints, hence known as break-bone fever. Other more severe manifestations of dengue infection, such as dengue hemorrhagic fever (DHF) and dengue shock syndrome (DSS) can be fatal if unrecognized and not properly treated in a timely manner. The prevalence of this disease has grown in recent decades and is now endemic in more than 100 countries [6].

The World Health Organization lists Dengue as a tropical neglected disease with other parasitic and bacterial infections [7], [8]. The cumulative reported numbers of dengue cases in 2012 showcase the devastating impact of dengue on human health around the world: 262,542 cases (data up to week ending 14 Nov) in the western pacific region [9] and 992,285 cases (data up to epidemiological week 43) in the Americas [10]. Furthermore, the economic cost of dengue in the Americas reached US$ 2.1 billion per year [11] and in Puerto Rico the aggregate annual cost averaged US$ 38.7 million [12]. A clear picture of the dengue global geographic expansion is of consequence, because vector control remains as the main resource for disease containment, since the leading vaccine candidate was only 30 percent effective in its first large clinical trial [13].

RNA virus molecular evolution is fast and occurs at approximately the same temporal scale of the ecological processes that shape their diversity [14]–[16]. This sanctions the use of Bayesian methods to phylodynamics [17], an approach that infer the origins and distribution of different viral strains, which are particularly useful for healthcare agents that may prefer maps and trends rather than raw numbers to communicate health emergencies [18], [19].

Despite high-endemicity of dengue viruses in tropical and subtropical countries, relatively few studies document viral dynamics and patterns of spread among human populations [20]–[25]. For DENV-1, Chen and Vasilakis [26] unrevealed biogeographical patterns of dispersal using 1812 strains based and taxa associations from the topology of tree. That study highlighted the role of Southeast Asian countries as sources of dengue epidemics and suggested a pattern of evolution radiating around spatially-defined geographic clades. Moreover, Allicock *et al*. [27] reconstructed the spatiotemporal spread of the virus in the Americas with 109 genotype V strains and documented the Lesser Antilles as the geographic location of ancestral infection. Therefore, in order to expand the knowledge on DENV-1 molecular epidemiology, by moving beyond the single place/time paradigm phylogenetic approach, bringing together a large number of sequences and providing a statistic comprehensive description on the genetic movement and distribution, the aim of this study was to reconstruct the temporal and spatial phylodynamics of different genotypes of DENV-1 and to stress its importance for control.

## Materials and Methods

### Sequence Data

Complete envelope sequences (E gene) of DENV-1 with known time (year) and geographical locality (country) of isolation were retrieved from GenBank (1583 envelope gene sequences from 45 distinct geographic locations, data up to Jan 2009). A gene approach was preferred over a genomic one because of high computational demands posed by model-based algorithms when handling larger sequences and also because much more sequences are available for that gene, especially for the old strains, providing a less biased dataset. However, the envelope is a major determinant of tropism and the primary target of virus-neutralizing antibodies and several previous studies have demonstrated its contribution to DENV phylogenetics [28]. Recombinant clones were removed for our analysis and pairwise homoplasy index (PHI) calculations did not show evidence for recombination in the final dataset (*p* = 0.2224) [29]. Sequences were aligned using Muscle software [30], [31] followed by visual inspection and manual editing with the Se-Al v2.0 program (http://tree.bio.ed.ac.uk/software/seal/).

### Genotyping

**Figure 1:**
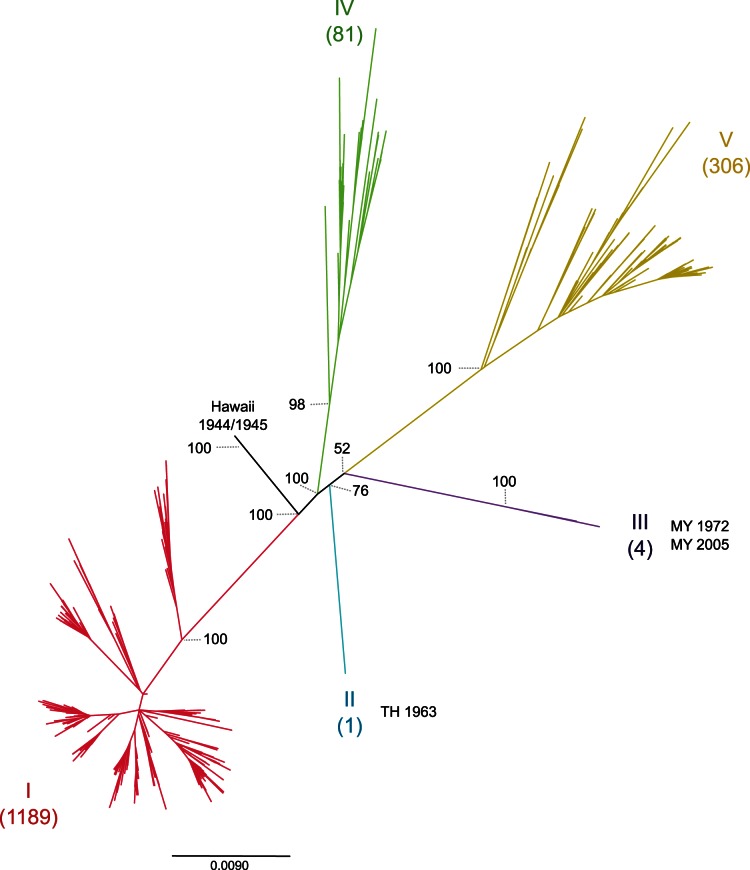
Evolutionary history of DENV-1. The phylogeny was inferred using a Maximum likelihood analysis of 1583 sequences. Because of the large number of isolates branch tips were removed. Numbers correspond to bootstrapping values.

In order to classify taxa and to create datasets for each genotype a Maximum likelihood (ML) tree was inferred using Garli v2.0 software and the 1583 E sequences [32]; bootstrapping values for branches were estimated with 100 non-parametric replicates This resulted in 1189 strains from Genotype I, one from Genotype II, four from Genotype III, 81 from Genotype IV and 306 from genotype V (Figure 1). Due to the large number of sequences available for Genotype I (773 being isolates from Vietnam), monophyletic groups with sequences from a single country were collapsed to include only the oldest taxon, the most recent and one halfway taxa (If there were several sequences at each of these time point, it was randomly selected). This procedure allowed the creation of a genotype I down sampled data set (180 taxa) that was used hereafter and was minimally representative of all possible migratory events over time. Also, a down sampled dataset for DENV-1 was built to estimate evolutionary parameters for the serotype (this exploration using over 500 sequences was computationally impractical in a Bayesian framework) by selecting 30 sequences representative in time and space from genotypes I, IV and V and all the available sequences from genotypes II and III.

### Likelihood Mapping

The phylogenetic signal of all datasets (full and down-sampled) was investigated by means of the likelihood mapping analysis of 10 million random quartets using TreePuzzle software [33]. For each quartet the three possible unrooted trees were reconstructed following a ML approach under the GTR+G+I substitution model, selected under the Akaike information criterion with JModeltest software [34]. The results were plotted as a triangle with fully resolved quartets shown as dots near the vertices and the unresolved quartets in the center of the triangle. Consequently, the relative phylogenetic signal in a map is directly proportional to the percentage of the concentration of dots at the vertices.

### Bayesian Phylodynamics Reconstruction

The usefulness and justifications for studying dengue systematics and evolution using Bayesian-based methods are argued for elsewhere [35]–[39]. Tree topologies, model parameters, evolutionary rate and viral population size variation over time were co-estimated independently for the down-sampled DENV-1 (95 taxa) and genotypes I (180 taxa), IV (81 taxa) and V(306 taxa) datasets with an uncorrelated log-normal relaxed clock model and the MCMC method implemented in the Beast package version 1.7 [35]. Genotypes II and III were not included because they both had less than five available sequences. A Bayesian skyline plot (an approach with no dependence on a pre-specified parametric model of demographic history) was used as a coalescent prior during the estimation over time of the change in *Ne.g* (the product of the effective population size and the generation length in years) [36]. The *Ne.g* parameter is a surrogate for the variation of viral population size and approximates the number of new infections. Importantly, *Ne.g* estimated from viral genealogies was shown to be proportional to the number of dengue cases in real epidemics [23]. The MCMC analysis was run four times and until convergence (with a minimum of 24 million generations sampling every 8000). We measured MCMC convergence by estimating the effective sampling size (ESS), after a 20% burn-in, using Tracer software version 1.5 (http://tree.bio.ed.ac.uk/software/tracer/). We estimated uncertainty as 95% high probability densities (95% HPD. All parameters showed ESS values >100. The obtained topologies were summarized in a maximum clade credibility (MCC) tree.

### Bayesian Phylogeographic Analyses

The Beast program allows for an ancestral reconstruction of discrete states where the spatial diffusion of the time scaled genealogy is modeled as a continuous-time Markov chain (CTMC) process over discrete sampling locations [37]. We used the Bayesian stochastic search variable selection (BSSVS) approach, which assumes exchange rates in the CTMC to be zero with some prior probability, to find the most parsimonious set of rates explaining the diffusion process along the phylogenies for each genotype. We use the software Spread v1.0.4 [38] to visualize diffusion rates over time: each location was represented by two capital letters using the ISO 3166-1 alpha-code and coordinates corresponded to the centroids of each country; a Bayes factor (BF) test was run to identify the rates contributing to the migration path [37] and all rates yielded a substantial BF (>3), and rates with the higher values (>20) were listed in Table S2. We also used the BayesTraits software package (available at http://www.evolution.rdg.ac.uk/) to reconstruct, under a maximum likelihood approach, the viral migration rates among major geographical areas (coded as discrete multistate traits: Africa/Arabian Peninsula, Central America, East Asia, Indian Ocean, Indochina, Maritime Southeast Asia, Micronesia/Polynesia, South America and The Caribbean). These analyses were done reconstructing character state changes onto representative sample of trees collected during the stationary phase of the MCMC runs [39]. The inferred rates of change among character states were taken as surrogates for migration rates among main areas (nodes) and incorporated as attributes in a network analysis. The importance of each area for DENV-1 global spread was evaluated by estimating the betweenness centrality (BC) using the Cytoscape 2.8.3 program [40]. BC was chosen because it informs on the number of shortest paths from all vertices to all others that pass through a node in a network. Therefore, it is a more informative measure of global topologic importance of a node than connectivity (which indicates the number of local connections of a node irrespective of its neighbor’s connectivity properties) [41].

## Results and Discussion

### Evolutionary History of DENV1

We retrieved DENV-1 envelope sequences from GenBank (Table S1 lists the accession numbers) and estimated the phylogenetic noise in each data set. We assumed that all of them contained phylogenetic signal (Figure S1) since none of them had more than 30% of unresolved quartets (i.e., noise), further supporting the adequacy of the phylogenetic inference methods that followed.

The Full DENV-1 dataset used in this study came from 45 geographic distinct locations, ranging from Hawaii (EU848545 being a pooled serum from six patients in 1944) to a single 2009 isolate from Cambodia. The ML tree (Figure 1) showed the five genotypes previously proposed [28], [42]: (*i*) Genotype I included strains from Southeast Asia and East Africa, (*ii*) Genotype II included a strain from Thailand collected in 1963, (*iii*) Genotype III included the sylvatic strain collected in Malaysia and the strain FN825674 isolated in 2005, (*iv*) Genotype IV included strains from Southeast Asia, the West Pacific islands, Australia and a few from the Americas, (*v*) Genotype V represented all strains collected in the Americas, West Africa, and a limited number of strains collected from Asia. The two closely-related Hawaiian strains isolated in 1944 and 1945 had a 5.7% estimated sequence divergence when compared to Genotype I. Hence, we grouped them together following the canonical definition of a genotype: a monophyletic group of dengue viruses having no more than 6% sequence divergence among them (Table S3) [43].

**Figure 2:**
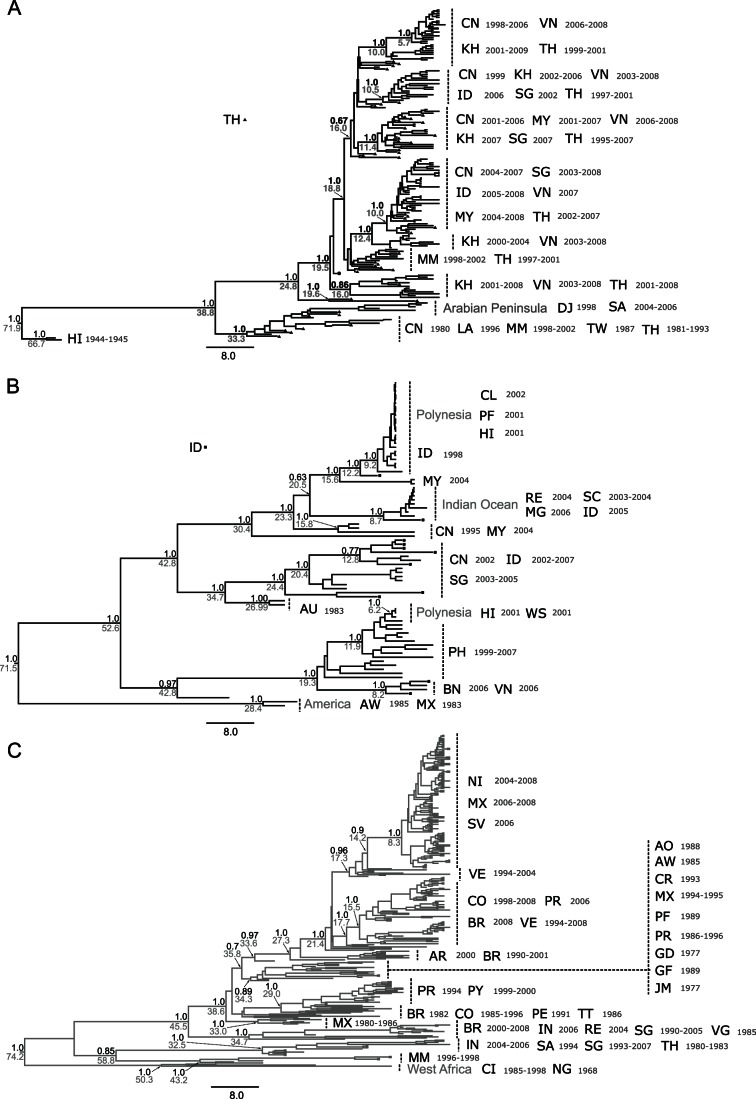
The Phylogenetic relationships of DENV-1 genotypes. Topologies correspond to Maximum Clade Credibility trees (MCC) for A) Genotype I, B) Genotype IV and C) Genotype V. Because of the large number of isolates branch tips were removed. Major geographical clades are indicated (ISO 3166 Codes). Black numbers correspond to posterior probability estimates and gray numbers correspond to the TMRCAs. Scale bar for years. Triangles represent Thailand isolates and squares represent Indonesia isolates.

The larger genotype datasets were used for phylogenetic reconstruction. Figure 2 shows the summarized the MCC trees for each genotype. There was no evident monophyly by country (Except for an Arabian clade within Genotype I) or by year of isolation and most clades had posterior probabilities values of 1.0. We argue that, even when studies showed monophyletic grouping by time and place for other serotypes [22], [44], in our case, the inclusion of more sequences within a spatiotemporal context revealed greater distribution and circulation of lineages among localities. For instance, well-sampled locations appeared throughout some topologies: Thailand appeared all over the genotype I tree, whilst strains from Indonesia appeared all over the genotype IV tree. This justifies the use of large samples to satisfactorily address viral diffusion.

### Rate of Nucleotide Substitution and TMRCA

The mean rate of nucleotide substitution was estimated using 95 taxa (30 sequences from Genotype I, IV and V; one sequence from genotype II and four sequences from genotype III) and resulted in 6.56×10−4 substitutions/site/year (Table 1). Rate values were compatible with those reported for dengue (6–8×10−4 substitutions/site/year [28] and related flaviviruses [45]–[47].

**Table 1:** Estimated DENV-1 substitutions rates and the time to the most recent common ancestor (TMRCA) estimated under a Bayesian Skyline coalescent tree prior.

Set	Number ofsequences	Rate (subs/site/year)	Coefficient of Variation	Rate 95% HPD	Node Age	95% HPD	TMRCA (Year)
DENV-1	95	^6.56×10−4^	^0.381^	^5.32×10−4–7.79×10−4^	109.17	91.20–129.75	1899
Genotype I	180	^9.90×10−4^	^0.408^	^8.75×10−4–1.11×10−3^	71.96	65.68–81.73	1937
Genotype IV	81	^9.07×10−4^	^0.906^	^6.11×10−4–1.26×10−3^	71.53	41.81–110.96	1936
Genotype V	306	^6.71×10−4^	^0.711^	^5.70×10−4–7.65×10−4^	73.41	56.73–93.20	1935

DENV-1 sequences appeared to evolve under a non-strict albeit clock-like manner because the estimated coefficient of variation under uncorrelated substitution rates was 0.381 (A value higher than zero supports the assumption of rate heterogeneity). A comparable value was obtained for DENV-4 [48]. Therefore, a relaxed clock model was used in the calculation of rates of substitution for each genotype separately. The estimated time to the most recent common ancestor (TMRCA) was 109 years (95% HPD 91.20–129.75) (Table 1). Remarkably, the TMRCA for each genotype was similar (∼72.5 years).

### DENV-1 Phylogeography

**Figure 3:**
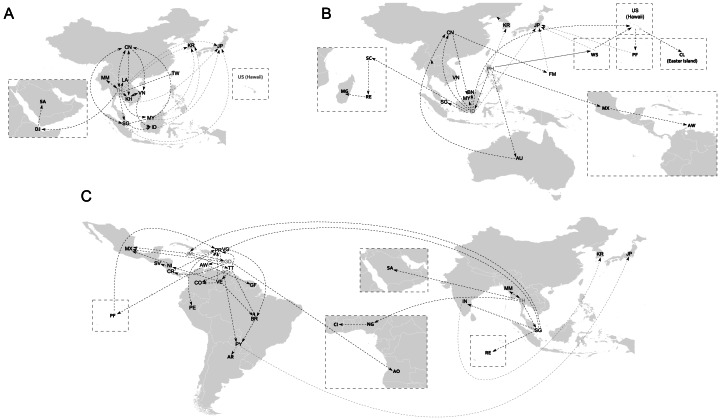
The Spread of DENV-1 genotypes. The likely dispersion routes were estimated from a discrete diffusion phylogeography process for A) Genotype I, B) Genotype IV and C) Genotype V. Dotted lines represent plausible migratory paths. Gray acronyms represent the apparent source of origin for each genotype (ISO 3166 Codes). Grey lines represent inferred introductions by studies with diseased travelers. A location-annotated MCC to KML for visualization over time is available for Google Earth as supporting material.

We used a discrete phylogeography analysis to provide a somewhat clear picture of its phylogeography: the interactive KML files (Dataset S1, S2 and S3) display the inferred distribution history over time for each genotype and Figure 3 presents a summary of the plausible migratory paths. An assumption of the discrete model of location change is that ancestral viruses necessarily reside at only the sampled locations of the extant viruses, hence, the methods describe the underlying spatial dynamics more accurately as the sampling density increase [37]. The DENV-1 sequence data studied, including the down-sampled genotype I, were diverse across a larger set of geographic locations. However, we are aware that most countries experiencing dengue epidemics do not have published sequences nor the sampling is homogeneous over time. Therefore, given the limitations and the uncertainty associated with each state reconstruction, in some cases instead of pointing to a specific location, we referred to the region to which it belongs.

### Distribution of Genotype I in Asia

Genotype I was restricted to Asia with an incursion into the Arabian Peninsula (KML S1 and Figures 2A and 3A; full MCC tree file available upon request). Taxa from the same country and year grouped in different clades. For example, we identified at least four different paraphyletic Cambodian clades associated with lineages from Vietnam. Duong *et al.* [49] also reported distinct Cambodian lineages and Raghwani *et al.* [50] reported that Vietnam received Cambodian lineages on different times. Altogether, these findings evidence a scenario where multiple lineages circulated in the same spatiotemporal context.

Hawaii appeared as the source of genotype I (state probability 0.83) but we argue that this result is biased because Hawaiian sequences are the oldest available so far: before and during World War II, dengue frequently occurred in US military personnel in Asia and the South Pacific, and dengue introduction into Honolulu occurred 1943 due to infected commercial airline pilots from the South Pacific [51]. Furthermore, the DENV-1 reference strain Mochizuki was isolated in 1943 in Japan [52]. The dominant location throughout the phylogeny and hence the more likely hub of diffusion is Thailand and together with its neighbors represent the most likely genotype I source. Actually, over the past decades, the number of cases increased steadily in that region [53].

### Distribution of Genotype IV in Asia and the Pacific

Genotype IV was found in Asia and the Pacific with an incursion into the Indian Ocean and the Americas (KML S2 and Figures 2B and 3B); Australian sequences also clustered within this genotype. Our findings suggest that the Philippines were probably the ancestral area for this genotype (state probability 0.14, other states with probabilities <0.07) and the source for both, one of the introductions into the Pacific and for the incursion into the Americas. Even so, the maritime country of Indonesia, the dominant location throughout the phylogeny, was the likely source for the spreading all over the region and into the Indian Ocean countries.

The Indian Ocean and Polynesians clades suggested that lineages diverged considerably for sufficient time after introduction, perhaps due to its geographical distance. Furthermore, their short branch lengths suggest rapid dissemination of viruses, possibly facilitated by vector competence [54] and host population susceptibility. We also found that strains from French Polynesia were associated with both, genotypes IV and V (Figure 2C). These findings are in line with phylogenetic studies that suggested multiple introductions of viruses from different genotypes into the Pacific [55].

When comparing Asian genotypes (Genotypes I and IV) it seems that genotype I distributes mostly over Indochina, while genotype IV distributes mostly over the Maritime Southeast Asia and Pacific islands, such as the Federated States of Micronesia (FM). Remarkably, some countries have reported different genotypes during the same outbreak (*e.g.*, Indonesia, Singapore and Vietnam), which begs the question of a possible role of intra-genotype competition impacting on genetic DENV diversity as suggested for Asian DENV-4 genotypes [24]. Further studies are crucial because about 400,000 cases of severe dengue, a leading cause of childhood mortality, are reported yearly in Southeast Asia with Thailand and Indonesia leading in number of cases [56].

While dengue is more prevalent in Southeast Asia and the Western Pacific region, other Asian countries also experienced DENV-1 outbreaks. For example, Guangdong ^_^ China’s most populated province ^_^ experienced outbreaks by DENV-1 since 1990 [57], [58]. In our study, samples from China obtained from 1993 to 2002 were classified as genotype IV, while the oldest Chinese sequence from 1980 and the most recent ones from 1998 to 2006 were classified as genotype I. Our results, as those of Chen [57], also suggested that Singapore, Thailand and Vietnam were plausible sources of DENV-1 for China, Cambodia and Malaysia. Moreover, studies with symptomatic travelers revealed that dengue DENV-1 strains are frequently introduced into Japan and Korea (90% of cases) from Southeast Asia [59], [60] where the disease has been an important health problem since international travel became popular in developing countries.

### Distribution of Genotype V in the Americas and the Caribbean

Genotype V comprises most American and Caribbean isolates and is wide distribution suggest *in situ* evolution among a susceptible human population after its introduction from Asia (KML S3 and Figures 2C and 3C)**.** Clades belonged mostly to a specific region (Caribbean, South America or Central America). Carvalho et al. [56] showed the existence of various country-specific DENV-1 founder effects, which may have fostered geographical structuring in the Americas. We argue that the lack of a larger number of samples from most countries might be masking a greater exchange of virus as the ones displayed by Puerto Rico, Venezuela and Brazil.

We inferred the introduction of genotype V into the Caribbean around 1971 and of genotype IV into Central America around 1979 (in line with Allicock et al. [27] estimates). Extensive epidemic outbreaks of DENV-1 were documented in 1977 in Puerto Rico, northeastern Mexico and the United States (Texas) [61], [62]. The low representation of Genotype IV over the region might be explained by cross-protection of immunity granted by the earlier exposure to Genotype V.

Our inferences showed that a strain from Southeast Asia (represented by Singapore with state probability 0.14 followed by Thailand with state probability 0.12) was related to the lineage introduced into the Caribbean (represented by Jamaica with state probability 0.22 followed by Grenade and Trinidad and Tobago with states probabilities 0.21 and 0.19 respectively) and therefore reinforces the notion that this region was an epicenter of the disease. The virus then moved into Grenada and Mexico and eventually to neighbor countries. We documented a second introduction, also from Singapore (state probability 0.23), into the British Virgin Islands and from there (state probability 0.76) to Brazil. The Pan American Health Organization attributed 20% of the Dengue cases in the Americas to DENV-1 during 1995–2007 and together with DENV-2 were the most frequent serotypes during the 1990s [63]. DENV-1 was detected for the first time in Brazil in 1981 and became a nationwide public health issue following the 1986 outbreak [64]. Outbreaks have been reported in Paraguay since 1988 and in Argentina from 1999 to 2000 in people traveling from Paraguay [65], [66].

### DENV-1 Distribution in Africa, Middle East and Europe

We documented DENV-1 dispersal into Africa, the Indian Ocean and the Arabian Peninsula. Different countries in Africa reported outbreaks, such as Djibouti, Côte d´Ivoire, Gabon, Kenya, Madagascar, Mayotte, Senegal, Sudan and Zanzibar [67], [68]. Nonetheless, conditions in African countries preclude more frequent diagnostic testing and adequate surveillance, leading to dengue sub-notification that hampers a better understanding of the history and spread dynamics of DENV-1 in that continent. Critically, Africa’s role in the worldwide spread of dengue cannot be underestimated. Domingo *et al.* [69] reported DENV-1 in samples from infected European travelers returning from dengue endemic areas from 2002 to 2008: Strains imported from Africa belonged to the three described genotypes, strains imported from Asia belonged to genotype I and IV and strains imported from Central America, the Caribbean and India belonged to genotype V.

### A comprehensive Overview of the Worldwide DENV-1 Transmission Network

**Figure 4:**
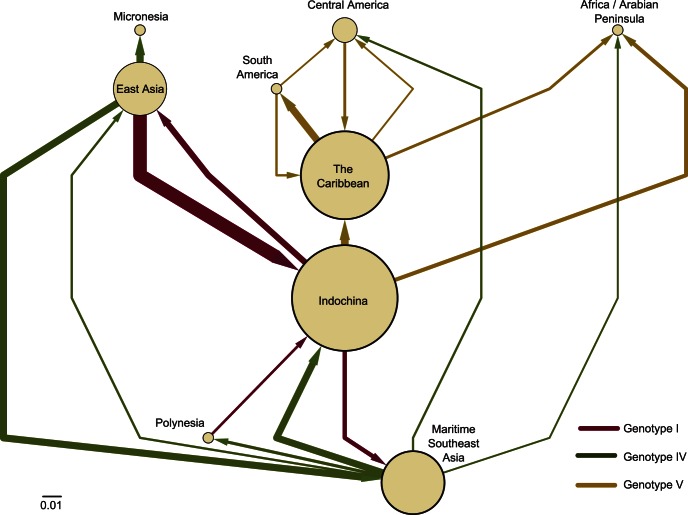
Network visualization of diffusion of DENV-1 genotypes. The analysis was done over major geographical areas and integrating migration rates among them as attributes. Thickness gauge at the bar scale for rates.

After obtaining a coarse picture of the spread of three main DENV-1 genotypes around the globe, we envisaged how the different world regions interconnect as a comprehensive web of transmission for the three main genotypes. Figure 4 depicts a spatially-adimensional, directed DENV-1 transmission network among major geographical areas. Node diameter and position are shown proportional to betweenness centrality (BC), which informs on the importance of the node in the whole transmission network. The arrows among nodes represent highly supported routes, while their thicknesses are proportional to the rates of viral movement among regions. This modeling exercise encapsulates the pivotal role of Indochina in the global epidemiology and transmission dynamics of DENV-1, as shown by its higher BC and the role of Maritime Southeast Asia in the spread of for genotype IV in the Americas. Moreover, East Asia (represented by southern China provinces) had extensive viral exchanges of Asian genotypes while in other localities, like Africa and the Arabian Peninsula, strains are apparently only introduced. Another interesting feature of this network is its modularity, since the Caribbean (second highest BC value after Indochina) did act as an important hub, mainly to Latin America while not exchanging significantly with Asia. Nevertheless, we must exercise caution when drawing conclusion about these findings because of the inferential limitations imposed by uneven spatial sampling in general.

### Complex Demographic History of DENV-1 Genotypes

**Figure 5:**
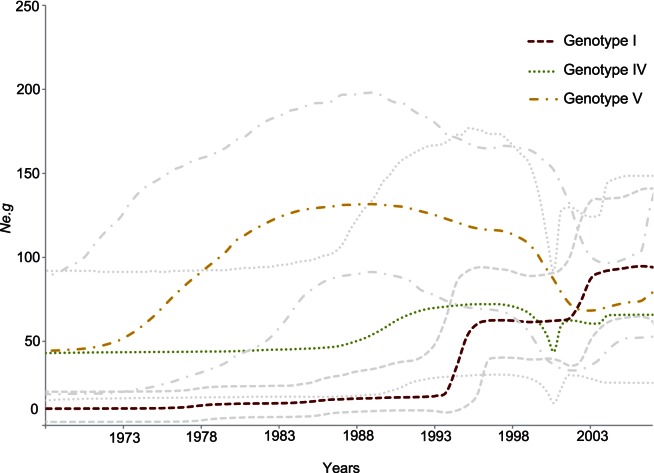
Demographic history for each genotype. Overlay of Bayesian skyline plot showing the *Ne.g.* (product of the effective population size and the generation length) for each DENV-1 genotypes. Ninety-five percent high probability densities (95% HPD) are showed as thin discontinuous lines around each mean plot.

We reconstructed the population dynamics through time of the three main genotypes (I, IV and V). Figure 5 shows each demographic history indicated by superimposed skyline plots of *Ne.g* that approximates the number of new infections over time. All plots had a maintenance phase until the 1970s when genetic diversity remained constant. The most conspicuous feature is the increase of oscillation in all skyline plots in the 1990’s.

Genotype I *Ne.g* exhibited two growing phases, one around 1994 and another one around 2002. Both phases were followed by stationary phases. Emerging lineages with epidemic potential, might explain this increase. For instance, Thai strains fell in multiples clades in the phylogeny (Figure 2A) and most of these fall into two different time periods: before 2001 and after 2002 coinciding with the second growing phase. Therefore, it seems that clade replacements occurred around those years and increased viral diversity. Crucially, a large outbreak occurred in Myanmar in 2001 [70] where DENV-1 displaced the other three serotypes.

Zhang *et al.* [71] documented DENV-1 clade replacements during the mid-1990s; events that coincided with the first growing phase. Studies showed that the replacements are associated with changes in serotype prevalence (a consequence of differential susceptibility to cross-reactive immune responses) and enhanced mosquito transmission [71], [72]. Recently, Yamanaka et al. [73] reported the displacement of Indonesian serotype 2, by DENV-1 Genotype I, with the proportion of severe cases increasing about three times.

Genotypes IV *Ne.g* grew steadily since 1988 and experienced a severe bottleneck around 2000 that reflect a possible limited availability of susceptible hosts: DENV-2 have was the prevalent serotype in the largest cities of Indonesia from 1998 to 2008 [73], [74] and also in the Philippines from 1995 to 2001 [75]. In addition, Asian DENV-4 increased incidence from 1996 to 2000 [24]. Nonetheless, the large outbreak over the Pacific during 2001 and 2002 that affected Palau, French Polynesia, Samoa, American Samoa, New Caledonia, Cook Islands, Tokelau, Wallis and Fortuna, Fiji and Solomon Islands and also reached Hawaii, Easter Island and Australia [76]–[78] might helped to overcome the bottleneck because of the number of new infections.

Genotype V *Ne.g* started to grew steadily since 1973, reached a zenith during the late 1980s and decline after 1988. The higher *Ne.g* values coincided with its ongoing distribution over the Americas and the Caribbean [79]. Moreover, the decline coincided with the introduction of DENV-3 genotype III into Panama in 1994, that spread throughout the Americas, displacing DENV-1 [80].

### Revisiting the Origins of DENV-1

Ecologic and socio-demographic changes of the 20th Century favored the emergence of dengue, particularly the end of World War II led to uncontrolled urbanization where mosquitoes vector reached high densities and facilitated dispersal of dengue among diverse geographic regions, and of adequate conditions for the emergence of DHF in Southeast Asia [28], [81]. Based on the oldest isolates, Mochizuki strain isolated in 1943 and the extant genotype II represented by Thailand isolated in 1963, we argue that DENV-1 evolved in Asia and later spread into Africa and the Americas [76]. Besides, Malaysia, is considered an area where all dengue serotypes evolved independently from a sylvatic ancestral lineage [82] and during a 2004 outbreak Teoh *et al.* documented a human DENV-1 strain that had >97% sequence similarity to the strain isolated from a sentinel monkey in 1972 [83]. Therefore, Southeast Asia still remains as an area sheltering a sylvatic mosquito transmission cycle.

Stoddard et al. [84] have shown that human movement is a critical, understudied behavioral component underlying the transmission dynamics of many vector-borne pathogens. Nonetheless, only a few studies address the issues of international travel that is responsible for the increasingly global widespread and complex distribution of the four DENV [50]. Wilder-Smith and Gubler [85] argued that epidemics, their seasonality, and oscillations over time are reflected by the epidemiology of dengue in travelers. Yet, the pattern of DEN-1 distribution presented here, seems to agree with that reported for DENV-4 in the Caribbean [86]. There, regional social and commercial activity relates to the exchange of viral strains, by means of the sporadic spread to remote regions where, if unimpeded by host immunity, it will result in regional epidemics until host immunity acts as a barrier to continued regional viral flow. This scenario is worsened by the only brief protection against heterologous infection with a different serotype setting the endemic nature of dengue in the tropical regions by spatial–temporal travelling waves of the dengue disease [87], [88].

At present, it seems likely that DENV activity will continue in tropical countries, as climate change, international travel, lack of vector control and urbanization, may all contribute to sustained epidemics and virus emergence and reemergence [89], [90]. As an example, outbreaks of dengue occurred in Hawaii by an unknown serotype in the 1840s, by DENV-1 genotype 1 in 1943 and 1944 and by different strains of DENV-1 genotype IV in 2001 [91], [92]. Once awareness’s practices related to the disease have demonstrated adequate prevalence of preventive practices against the disease [93], the knowledge of the spatial-temporal dynamics of DENV-1 provides an important starting point for control initiatives by policy-makers. Understanding movement will facilitate identification of key localities for transmission which may provide targets for mosquito surveillance intensification and improved disease prevention.

In sum, we have compiled much of the available DENV-1 sequence data to unveil some of the history of their distribution around the world. We also reconstructed the demographic history of the genotypes and highlighted how spread and viral diversity make control of dengue a challenging task. Further studies are mandatory because dengue epidemics occur as a result of the emergence of new lineages and dissemination. Our results also highlight the need to overcome non-uniform sampling from distinct locations, especially from Africa. Despite the fact that dengue is regularly reported in several important regions, national surveillance data still significantly underestimate the true burden of the disease [94].

## Supporting Information

Figure S1 **Likelihood mapping diagrams for each genotype dataset.** The relative phylogenetic signal is directly proportional to the percentage of the concentration of dots at the vertices.
(EPS)

Table S1 **List of GenBank sequences used in this study.**
(DOCX)

Table S2 **The significant connections at Bayes factor 20 to establish epidemiological linkage in viral phylogeographic histories.**
(DOCX)

Table S3 **Estimates of Net Evolutionary Divergence between groups of sequences for the complete DENV-1 dataset.** Analyses were conducted using the Maximum Composite Likelihood model. The rate variation among sites was modeled with a gamma distribution (shape parameter = 0.294).
(DOCX)

Dataset S1 **Interactive KML for Google Earth showing the reconstructed dispersal history over time for genotype I.**
(RAR)

Dataset S2 **Interactive KML for Google Earth showing the reconstructed dispersal history over time for genotype IV.**
(RAR)

Dataset S3 **Interactive KML for Google Earth showing the reconstructed dispersal history over time for genotype V.**
(RAR)
