# Supplementary material for: Worldwide Spread of Dengue Virus Type 1
Source: PLoS One. 2013 May 13;8(5):e62649. doi: 10.1371/journal.pone.0062649 (PMC3652851; doi:10.1371/journal.pone.0062649)
Supplement: Table S1 — List of GenBank sequences used in this study. (DOCX) [file pone.0062649.s002.docx]

**Table S1.** List of GenBank sequences used in this study.

| **Genotype** | **Country** | **Accession** | **Year** |
| --- | --- | --- | --- |
| I | US (HI) | EU848545 | 1944 |
| I | US (HI) | AF425619 | 1945 |
| III | TH | AF425629 | 1963 |
| I | TH | AY732378 | 1982 |
| I | KH | FJ744702 | 2006 |
| I | TH | FJ687433 | 2001 |
| I | TH | FJ687432 | 2001 |
| I | TH | FJ687431 | 2001 |
| I | TH | FJ687430 | 2001 |
| I | TH | FJ687429 | 2001 |
| I | TH | FJ687428 | 2001 |
| I | TH | FJ687427 | 2001 |
| I | TH | FJ687426 | 2001 |
| I | SG | GQ398255 | 2008 |
| I | VN | HM631850 | 2008 |
| I | VN | HM488256 | 2008 |
| I | KH | HM488255 | 2007 |
| I | KH | HM631853 | 2007 |
| I | KH | HM631852 | 2006 |
| I | VN | HM631851 | 2008 |
| I | VN | HM181970 | 2008 |
| I | VN | HM181969 | 2008 |
| I | VN | HM181968 | 2008 |
| I | VN | HM181967 | 2008 |
| I | VN | HM181966 | 2008 |
| I | VN | HM181965 | 2008 |
| I | VN | HM181964 | 2008 |
| I | VN | HM181963 | 2008 |
| I | VN | HM181962 | 2008 |
| I | VN | HM181961 | 2008 |
| I | VN | HM181960 | 2008 |
| I | KH | HM181959 | 2007 |
| I | KH | HM181958 | 2007 |
| I | KH | HM181957 | 2007 |
| I | KH | HM181956 | 2007 |
| I | KH | HM181955 | 2007 |
| I | KH | HM181954 | 2007 |
| I | KH | HM181953 | 2007 |
| I | KH | HM181952 | 2007 |
| I | KH | HM181951 | 2007 |
| I | KH | HM181950 | 2007 |
| I | KH | HM181949 | 2007 |
| I | KH | HM181948 | 2007 |
| I | KH | HM181947 | 2007 |
| I | KH | HM181946 | 2007 |
| I | KH | HM181945 | 2007 |
| I | KH | HM181944 | 2007 |
| I | KH | HM181943 | 2007 |
| I | KH | HM181942 | 2006 |
| I | KH | HM181941 | 2006 |
| I | KH | HM181940 | 2006 |
| I | KH | HM181939 | 2006 |
| I | KH | HM181938 | 2006 |
| I | KH | HM181937 | 2006 |
| I | KH | HM181936 | 2006 |
| I | DJ | AF298808 | 1998 |
| I | SG | FJ469909 | 2003 |
| I | SG | FJ469908 | 2003 |
| I | SG | FJ469907 | 2003 |
| I | SG | EU081262 | 2005 |
| I | SG | EU081254 | 2005 |
| I | SG | EU081281 | 2006 |
| I | SG | EU081280 | 2006 |
| I | SG | EU081279 | 2005 |
| I | SG | EU081278 | 2005 |
| I | SG | EU081277 | 2005 |
| I | SG | EU081276 | 2005 |
| I | SG | EU081275 | 2005 |
| I | SG | EU081274 | 2005 |
| I | SG | EU081273 | 2005 |
| I | SG | EU081272 | 2005 |
| I | SG | EU081271 | 2005 |
| I | SG | EU081270 | 2005 |
| I | SG | EU081269 | 2005 |
| I | SG | EU081268 | 2005 |
| I | SG | EU081267 | 2005 |
| I | SG | EU081266 | 2005 |
| I | SG | EU081265 | 2005 |
| I | SG | EU081264 | 2005 |
| I | SG | EU081263 | 2005 |
| I | SG | EU081261 | 2005 |
| I | SG | EU081260 | 2005 |
| I | SG | EU081259 | 2005 |
| I | SG | EU081257 | 2005 |
| I | SG | EU081256 | 2005 |
| I | SG | EU081255 | 2005 |
| I | SG | EU081253 | 2005 |
| I | SG | EU081252 | 2005 |
| I | SG | EU081251 | 2005 |
| I | SG | EU081250 | 2005 |
| I | SG | EU081249 | 2005 |
| I | SG | EU081248 | 2005 |
| I | SG | EU081247 | 2005 |
| I | SG | EU081246 | 2005 |
| I | SG | EU081245 | 2005 |
| I | SG | EU081244 | 2005 |
| I | SG | EU081243 | 2005 |
| I | SG | EU081242 | 2005 |
| I | SG | EU081241 | 2005 |
| I | SG | EU081240 | 2005 |
| I | SG | EU081239 | 2005 |
| I | SG | EU081238 | 2005 |
| I | SG | EU081237 | 2005 |
| I | SG | EU081236 | 2005 |
| I | SG | EU081235 | 2005 |
| I | SG | EU081234 | 2005 |
| I | SG | EU081233 | 2005 |
| I | SG | EU081232 | 2005 |
| I | SG | EU081231 | 2005 |
| I | SG | EU081230 | 2005 |
| I | SG | EU081229 | 2005 |
| I | SG | EU081228 | 2005 |
| I | SG | EU081227 | 2005 |
| I | SG | EU081226 | 2005 |
| I | TH | AY732483 | 1981 |
| I | TH | AY732482 | 2001 |
| I | TH | AY732481 | 1982 |
| I | TH | AY732480 | 1994 |
| I | TH | AY732479 | 2001 |
| I | TH | AY732478 | 1991 |
| I | TH | AY732477 | 1991 |
| I | TH | AY732475 | 1994 |
| I | KH | GU131926 | 2006 |
| I | KH | GU131925 | 2006 |
| I | KH | GU131923 | 2005 |
| I | KH | GU131922 | 2008 |
| I | KH | GU131921 | 2008 |
| I | KH | GU131920 | 2008 |
| I | KH | GU131919 | 2008 |
| I | KH | GU131895 | 2009 |
| I | KH | GU131894 | 2008 |
| I | KH | GU131893 | 2007 |
| I | KH | GU131892 | 2006 |
| I | KH | GU131891 | 2006 |
| I | KH | GU131890 | 2006 |
| I | KH | GU131889 | 2006 |
| I | KH | GU131888 | 2006 |
| I | KH | GU131887 | 2006 |
| I | KH | GQ868639 | 2006 |
| I | KH | GQ868637 | 2000 |
| I | KH | GQ868636 | 2008 |
| I | KH | GQ868635 | 2008 |
| I | KH | GQ868633 | 2008 |
| I | KH | GQ868632 | 2008 |
| I | KH | GQ868630 | 2006 |
| I | KH | GQ868619 | 2003 |
| I | KH | GQ868618 | 2003 |
| I | VN | GQ868615 | 2008 |
| I | VN | GQ868614 | 2008 |
| I | VN | GQ868613 | 2006 |
| I | VN | GQ868612 | 2006 |
| I | VN | GQ868611 | 2007 |
| I | VN | GQ868610 | 2007 |
| I | VN | GQ868609 | 2007 |
| I | VN | GQ868608 | 2007 |
| I | VN | GQ868607 | 2007 |
| I | VN | GQ868606 | 2006 |
| I | VN | GQ868605 | 2006 |
| I | VN | GQ199856 | 2006 |
| I | VN | GQ199855 | 2006 |
| I | VN | GQ199854 | 2006 |
| I | VN | GQ199853 | 2006 |
| I | VN | GQ199852 | 2006 |
| I | VN | GQ199851 | 2006 |
| I | VN | GQ199850 | 2006 |
| I | VN | GQ199849 | 2006 |
| I | VN | GQ199848 | 2006 |
| I | VN | GQ199847 | 2006 |
| I | VN | GQ199846 | 2006 |
| I | VN | GQ199845 | 2006 |
| I | VN | GQ199844 | 2006 |
| I | VN | GQ199843 | 2006 |
| I | VN | GQ199842 | 2006 |
| I | VN | GQ199841 | 2006 |
| I | VN | GQ199840 | 2006 |
| I | VN | GQ199839 | 2006 |
| I | VN | GQ199838 | 2005 |
| I | VN | GQ199837 | 2005 |
| I | VN | GQ199836 | 2005 |
| I | VN | GQ199835 | 2005 |
| I | VN | GQ199834 | 2005 |
| I | VN | GQ199833 | 2004 |
| I | VN | GQ199832 | 2003 |
| I | VN | GQ199831 | 2003 |
| I | VN | GQ199830 | 2003 |
| I | VN | GQ199829 | 2007 |
| I | VN | GQ199828 | 2007 |
| I | VN | GQ199827 | 2007 |
| I | VN | GQ199826 | 2007 |
| I | VN | GQ199825 | 2007 |
| I | VN | GQ199824 | 2007 |
| I | VN | GQ199823 | 2007 |
| I | VN | GQ199822 | 2007 |
| I | VN | GQ199821 | 2007 |
| I | VN | GQ199820 | 2007 |
| I | VN | GQ199819 | 2007 |
| I | VN | GQ199818 | 2007 |
| I | VN | GQ199817 | 2007 |
| I | VN | GQ199816 | 2007 |
| I | VN | GQ199815 | 2007 |
| I | VN | GQ199814 | 2007 |
| I | VN | GQ199813 | 2007 |
| I | VN | GQ199812 | 2007 |
| I | VN | GQ199811 | 2007 |
| I | VN | GQ199810 | 2007 |
| I | VN | GQ199809 | 2007 |
| I | VN | GQ199808 | 2007 |
| I | VN | GQ199807 | 2007 |
| I | VN | GQ199806 | 2007 |
| I | VN | GQ199805 | 2007 |
| I | VN | GQ199804 | 2007 |
| I | VN | GQ199803 | 2007 |
| I | VN | GQ199802 | 2007 |
| I | VN | GQ199801 | 2007 |
| I | VN | GQ199800 | 2007 |
| I | VN | GQ199799 | 2007 |
| I | VN | GQ199798 | 2007 |
| I | VN | GQ199797 | 2007 |
| I | VN | GQ199796 | 2007 |
| I | VN | GQ199795 | 2007 |
| I | VN | GQ199794 | 2007 |
| I | VN | GQ199793 | 2007 |
| I | VN | GQ199792 | 2007 |
| I | VN | GQ199791 | 2007 |
| I | VN | GQ199790 | 2007 |
| I | VN | GQ199789 | 2007 |
| I | VN | GQ199788 | 2007 |
| I | VN | GQ199787 | 2007 |
| I | VN | GQ199786 | 2007 |
| I | VN | GQ199785 | 2007 |
| I | VN | GQ199784 | 2007 |
| I | VN | GQ199783 | 2007 |
| I | VN | GQ199782 | 2007 |
| I | VN | GQ199781 | 2007 |
| I | VN | GQ199780 | 2007 |
| I | VN | GQ199779 | 2007 |
| I | VN | GQ199778 | 2007 |
| I | VN | GQ199777 | 2007 |
| I | VN | GQ199776 | 2007 |
| I | VN | GQ199775 | 2007 |
| I | VN | GQ199774 | 2007 |
| I | VN | GQ199773 | 2007 |
| I | VN | GQ199772 | 2006 |
| I | VN | GQ199771 | 2006 |
| I | CN | DQ193572 | 2004 |
| I | MM | AY726555 | 1998 |
| I | MM | AY726554 | 1998 |
| I | MM | AY726553 | 2002 |
| I | MM | AY726552 | 2002 |
| I | MM | AY726551 | 2001 |
| I | MM | AY726550 | 2001 |
| I | MM | AY726549 | 2001 |
| I | MM | AY713476 | 2001 |
| I | MM | AY713475 | 2001 |
| I | MM | AY713474 | 2001 |
| I | MM | AY708047 | 2001 |
| I | CN | AF350498 | 1980 |
| I | VN | GU131831 | 2008 |
| I | VN | GU131830 | 2008 |
| I | VN | GU131829 | 2008 |
| I | VN | GU131828 | 2008 |
| I | VN | GU131827 | 2008 |
| I | VN | GU131826 | 2008 |
| I | VN | GU131825 | 2008 |
| I | VN | GU131824 | 2008 |
| I | VN | GU131823 | 2008 |
| I | VN | GU131822 | 2008 |
| I | VN | GU131821 | 2008 |
| I | VN | GU131820 | 2008 |
| I | VN | GU131819 | 2008 |
| I | VN | GU131818 | 2008 |
| I | VN | GU131817 | 2008 |
| I | VN | GU131816 | 2008 |
| I | VN | GU131815 | 2008 |
| I | VN | GU131814 | 2008 |
| I | VN | GU131813 | 2008 |
| I | VN | GU131812 | 2008 |
| I | VN | GU131811 | 2008 |
| I | VN | GU131810 | 2008 |
| I | VN | GU131809 | 2008 |
| I | VN | GU131808 | 2008 |
| I | VN | GU131807 | 2008 |
| I | VN | GU131806 | 2008 |
| I | VN | GU131805 | 2008 |
| I | VN | GU131804 | 2008 |
| I | VN | GU131803 | 2008 |
| I | VN | GU131802 | 2008 |
| I | VN | GU131801 | 2008 |
| I | VN | GU131800 | 2008 |
| I | VN | GU131799 | 2008 |
| I | VN | GU131798 | 2008 |
| I | VN | GU131797 | 2008 |
| I | VN | GU131796 | 2008 |
| I | VN | GU131795 | 2008 |
| I | VN | GU131794 | 2008 |
| I | VN | GU131793 | 2008 |
| I | VN | GU131792 | 2008 |
| I | VN | GU131791 | 2008 |
| I | VN | GU131790 | 2008 |
| I | VN | GU131789 | 2008 |
| I | VN | GU131788 | 2008 |
| I | VN | GU131787 | 2008 |
| I | VN | GU131786 | 2008 |
| I | VN | GU131785 | 2008 |
| I | VN | GU131784 | 2008 |
| I | VN | GU131783 | 2008 |
| I | VN | GU131782 | 2008 |
| I | VN | GU131781 | 2008 |
| I | VN | GU131780 | 2008 |
| I | VN | GU131779 | 2008 |
| I | VN | GU131778 | 2008 |
| I | VN | GU131777 | 2008 |
| I | VN | GU131776 | 2008 |
| I | VN | GU131775 | 2008 |
| I | VN | GU131774 | 2008 |
| I | VN | GU131773 | 2008 |
| I | VN | GU131772 | 2008 |
| I | VN | GU131771 | 2008 |
| I | VN | GU131770 | 2008 |
| I | VN | GU131769 | 2008 |
| I | VN | GU131768 | 2008 |
| I | VN | GU131767 | 2008 |
| I | VN | GU131766 | 2008 |
| I | VN | GU131765 | 2008 |
| I | VN | GU131764 | 2008 |
| I | VN | GU131763 | 2008 |
| I | VN | GU131762 | 2008 |
| I | VN | GU131761 | 2008 |
| I | VN | GU131760 | 2008 |
| I | VN | GU131759 | 2008 |
| I | VN | GU131758 | 2008 |
| I | VN | GU131757 | 2008 |
| I | VN | GU131756 | 2008 |
| I | VN | GU131755 | 2008 |
| I | VN | GU131754 | 2008 |
| I | VN | GU131753 | 2008 |
| I | VN | GU131752 | 2008 |
| I | VN | GU131751 | 2008 |
| I | VN | GU131750 | 2008 |
| I | VN | GU131749 | 2008 |
| I | VN | GU131748 | 2008 |
| I | VN | GU131747 | 2008 |
| I | VN | GU131746 | 2008 |
| I | VN | GU131745 | 2008 |
| I | VN | GU131744 | 2008 |
| I | VN | GU131743 | 2008 |
| I | VN | GU131742 | 2008 |
| I | VN | GU131741 | 2008 |
| I | VN | GU131740 | 2008 |
| I | VN | GU131739 | 2008 |
| I | VN | GU131738 | 2008 |
| I | VN | GU131737 | 2008 |
| I | VN | GU131736 | 2008 |
| I | VN | GU131735 | 2008 |
| I | VN | GU131734 | 2008 |
| I | VN | GU131733 | 2008 |
| I | VN | GU131732 | 2008 |
| I | VN | GU131731 | 2008 |
| I | VN | GU131730 | 2008 |
| I | VN | GU131729 | 2008 |
| I | VN | GU131728 | 2008 |
| I | VN | GU131727 | 2008 |
| I | VN | GU131726 | 2008 |
| I | VN | GU131725 | 2008 |
| I | VN | GU131724 | 2008 |
| I | VN | GU131723 | 2008 |
| I | VN | GU131722 | 2008 |
| I | VN | GU131721 | 2008 |
| I | VN | GU131720 | 2008 |
| I | VN | GU131719 | 2008 |
| I | VN | GU131718 | 2008 |
| I | VN | GU131717 | 2008 |
| I | VN | GU131716 | 2008 |
| I | VN | GU131715 | 2008 |
| I | VN | GU131714 | 2008 |
| I | VN | GU131713 | 2008 |
| I | VN | GU131712 | 2008 |
| I | VN | GU131711 | 2008 |
| I | VN | GU131710 | 2008 |
| I | VN | GU131709 | 2008 |
| I | VN | GU131708 | 2008 |
| I | VN | GU131707 | 2008 |
| I | VN | GU131706 | 2008 |
| I | VN | GU131705 | 2008 |
| I | VN | GU131704 | 2008 |
| I | VN | GU131703 | 2008 |
| I | VN | GU131702 | 2008 |
| I | VN | GU131701 | 2008 |
| I | VN | GU131700 | 2008 |
| I | VN | GU131699 | 2008 |
| I | VN | GU131698 | 2008 |
| I | VN | GU131697 | 2008 |
| I | VN | GU131696 | 2008 |
| I | VN | GU131695 | 2008 |
| I | VN | GU131694 | 2008 |
| I | VN | GU131693 | 2008 |
| I | VN | GU131692 | 2008 |
| I | VN | GU131691 | 2008 |
| I | VN | GU131690 | 2008 |
| I | VN | GU131689 | 2008 |
| I | VN | GU131688 | 2008 |
| I | VN | GU131687 | 2008 |
| I | VN | GU131686 | 2008 |
| I | VN | GU131685 | 2008 |
| I | VN | GU131684 | 2008 |
| I | VN | GU131683 | 2008 |
| I | VN | GU131682 | 2008 |
| I | VN | GU131681 | 2008 |
| I | VN | GU131680 | 2008 |
| I | VN | GU131679 | 2008 |
| I | VN | GU131678 | 2008 |
| I | CN | AY835999 | 2004 |
| I | VN | FJ432749 | 2007 |
| I | VN | FJ432748 | 2007 |
| I | VN | FJ432747 | 2007 |
| I | VN | FJ432746 | 2007 |
| I | VN | FJ432745 | 2007 |
| I | VN | FJ432744 | 2007 |
| I | VN | FJ432742 | 2007 |
| I | VN | FJ432740 | 2007 |
| I | VN | FJ432739 | 2007 |
| I | VN | FJ432738 | 2007 |
| I | VN | FJ432737 | 2007 |
| I | VN | FJ432736 | 2007 |
| I | VN | FJ432735 | 2007 |
| I | VN | FJ432734 | 2007 |
| I | VN | FJ432733 | 2007 |
| I | VN | FJ432732 | 2007 |
| I | VN | FJ432730 | 2007 |
| I | VN | FJ432729 | 2007 |
| I | VN | FJ432727 | 2007 |
| I | VN | FJ432725 | 2007 |
| I | VN | FJ432723 | 2007 |
| I | VN | FJ432719 | 2007 |
| I | VN | FJ410289 | 2007 |
| I | VN | FJ410287 | 2006 |
| I | VN | FJ410286 | 2008 |
| I | VN | FJ410285 | 2008 |
| I | VN | FJ410284 | 2008 |
| I | VN | FJ410283 | 2008 |
| I | VN | FJ410282 | 2008 |
| I | VN | FJ410281 | 2008 |
| I | VN | FJ410280 | 2008 |
| I | VN | FJ410279 | 2008 |
| I | VN | FJ410278 | 2008 |
| I | VN | FJ410277 | 2008 |
| I | VN | FJ410276 | 2008 |
| I | VN | FJ410275 | 2008 |
| I | VN | FJ410274 | 2008 |
| I | VN | FJ410273 | 2008 |
| I | VN | FJ410272 | 2008 |
| I | VN | FJ410270 | 2008 |
| I | VN | FJ410269 | 2008 |
| I | VN | FJ410268 | 2008 |
| I | VN | FJ410267 | 2008 |
| I | VN | FJ410266 | 2008 |
| I | VN | FJ410265 | 2008 |
| I | VN | FJ410264 | 2008 |
| I | VN | FJ410263 | 2008 |
| I | VN | FJ410262 | 2008 |
| I | VN | FJ410261 | 2008 |
| I | VN | FJ410260 | 2008 |
| I | VN | FJ410258 | 2008 |
| I | VN | FJ410257 | 2008 |
| I | VN | FJ410256 | 2008 |
| I | VN | FJ410255 | 2008 |
| I | VN | FJ410254 | 2008 |
| I | VN | FJ410253 | 2007 |
| I | VN | FJ410252 | 2007 |
| I | VN | FJ410251 | 2008 |
| I | VN | FJ410250 | 2007 |
| I | VN | FJ410249 | 2008 |
| I | VN | FJ410248 | 2008 |
| I | VN | FJ410247 | 2008 |
| I | VN | FJ410246 | 2008 |
| I | VN | FJ410245 | 2008 |
| I | VN | FJ410244 | 2008 |
| I | VN | FJ410243 | 2008 |
| I | VN | FJ410242 | 2008 |
| I | VN | FJ410240 | 2008 |
| I | VN | FJ410239 | 2008 |
| I | VN | FJ410238 | 2008 |
| I | VN | FJ410236 | 2008 |
| I | VN | FJ410235 | 2008 |
| I | VN | FJ410234 | 2007 |
| I | VN | FJ410232 | 2007 |
| I | VN | FJ410231 | 2007 |
| I | VN | FJ410230 | 2008 |
| I | VN | FJ410227 | 2007 |
| I | VN | FJ410226 | 2008 |
| I | VN | FJ410225 | 2007 |
| I | VN | FJ410222 | 2007 |
| I | VN | FJ410220 | 2008 |
| I | VN | FJ410218 | 2007 |
| I | VN | FJ410216 | 2007 |
| I | VN | FJ410214 | 2008 |
| I | VN | FJ410213 | 2008 |
| I | VN | FJ410212 | 2007 |
| I | VN | FJ410211 | 2007 |
| I | VN | FJ410210 | 2007 |
| I | VN | FJ410209 | 2007 |
| I | VN | FJ410207 | 2007 |
| I | VN | FJ410206 | 2007 |
| I | VN | FJ410205 | 2007 |
| I | VN | FJ410204 | 2007 |
| I | VN | FJ410203 | 2007 |
| I | VN | FJ410201 | 2007 |
| I | VN | FJ410199 | 2007 |
| I | VN | FJ410198 | 2007 |
| I | VN | FJ410197 | 2007 |
| I | VN | FJ410196 | 2007 |
| I | VN | FJ410194 | 2007 |
| I | VN | FJ410192 | 2008 |
| I | VN | FJ410191 | 2008 |
| I | VN | FJ390388 | 2007 |
| I | VN | FJ390386 | 2007 |
| I | VN | FJ390383 | 2007 |
| I | VN | FJ390382 | 2007 |
| I | VN | FJ390381 | 2007 |
| I | VN | FJ373305 | 2006 |
| I | VN | FJ373298 | 2007 |
| I | VN | FJ373297 | 2007 |
| I | VN | FJ373296 | 2006 |
| I | VN | FJ205884 | 2007 |
| I | VN | FJ205883 | 2007 |
| I | VN | FJ205882 | 2007 |
| I | VN | FJ205881 | 2007 |
| I | VN | FJ205876 | 2007 |
| I | VN | FJ182036 | 2007 |
| I | VN | FJ182035 | 2007 |
| I | VN | FJ182034 | 2007 |
| I | VN | FJ182033 | 2007 |
| I | VN | FJ182032 | 2007 |
| I | VN | FJ182031 | 2007 |
| I | VN | FJ182030 | 2007 |
| I | VN | FJ182029 | 2007 |
| I | VN | FJ182028 | 2007 |
| I | VN | FJ182027 | 2007 |
| I | VN | FJ182026 | 2007 |
| I | VN | FJ182025 | 2007 |
| I | VN | FJ182024 | 2007 |
| I | VN | FJ182023 | 2007 |
| I | VN | FJ182022 | 2007 |
| I | VN | FJ182021 | 2007 |
| I | VN | FJ182020 | 2007 |
| I | VN | FJ182019 | 2007 |
| I | VN | FJ182018 | 2006 |
| I | VN | FJ182003 | 2007 |
| I | VN | FJ024472 | 2007 |
| I | VN | FJ024464 | 2007 |
| I | VN | FJ024463 | 2007 |
| I | VN | FJ024462 | 2007 |
| I | VN | FJ024460 | 2007 |
| I | VN | FJ024459 | 2007 |
| I | VN | FJ024457 | 2007 |
| I | VN | FJ024456 | 2007 |
| I | VN | FJ024455 | 2007 |
| I | VN | FJ024453 | 2007 |
| I | VN | FJ024451 | 2007 |
| I | VN | FJ024450 | 2007 |
| I | VN | FJ024449 | 2007 |
| I | VN | FJ024448 | 2007 |
| I | VN | FJ024447 | 2007 |
| I | VN | FJ024446 | 2007 |
| I | VN | FJ024445 | 2007 |
| I | VN | FJ024444 | 2006 |
| I | VN | FJ024443 | 2007 |
| I | VN | FJ024442 | 2007 |
| I | VN | FJ024441 | 2007 |
| I | VN | FJ024440 | 2007 |
| I | VN | FJ024439 | 2007 |
| I | VN | FJ024438 | 2007 |
| I | VN | FJ024437 | 2007 |
| I | VN | FJ024436 | 2007 |
| I | VN | FJ024435 | 2007 |
| I | VN | FJ024434 | 2007 |
| I | VN | FJ024433 | 2007 |
| I | VN | FJ024432 | 2007 |
| I | VN | FJ024431 | 2007 |
| I | VN | FJ024430 | 2007 |
| I | VN | FJ024429 | 2007 |
| I | VN | FJ024428 | 2007 |
| I | VN | FJ024427 | 2007 |
| I | VN | FJ024426 | 2007 |
| I | VN | FJ024425 | 2007 |
| I | VN | EU726782 | 2007 |
| I | VN | EU726781 | 2007 |
| I | VN | EU726780 | 2007 |
| I | VN | EU726779 | 2007 |
| I | VN | EU726778 | 2007 |
| I | VN | EU726777 | 2007 |
| I | VN | EU687251 | 2007 |
| I | VN | EU687247 | 2006 |
| I | VN | EU677178 | 2007 |
| I | VN | EU677177 | 2007 |
| I | VN | EU677176 | 2007 |
| I | VN | EU677175 | 2007 |
| I | VN | EU677174 | 2007 |
| I | VN | EU677173 | 2007 |
| I | VN | EU677172 | 2007 |
| I | VN | EU677171 | 2007 |
| I | VN | EU677170 | 2007 |
| I | VN | EU677169 | 2007 |
| I | VN | EU677168 | 2007 |
| I | VN | EU677167 | 2007 |
| I | VN | EU677166 | 2007 |
| I | VN | EU677165 | 2007 |
| I | VN | EU677164 | 2007 |
| I | VN | EU677163 | 2007 |
| I | VN | EU677162 | 2007 |
| I | VN | EU677161 | 2007 |
| I | VN | EU677160 | 2007 |
| I | VN | EU677159 | 2007 |
| I | VN | EU677158 | 2007 |
| I | VN | EU677157 | 2007 |
| I | VN | EU677156 | 2007 |
| I | VN | EU677155 | 2007 |
| I | VN | EU677154 | 2007 |
| I | VN | EU677153 | 2007 |
| I | VN | EU677152 | 2007 |
| I | VN | EU677151 | 2007 |
| I | VN | EU677150 | 2007 |
| I | VN | EU677140 | 2007 |
| I | VN | EU677139 | 2007 |
| I | VN | EU660397 | 2006 |
| I | VN | EU660396 | 2006 |
| I | VN | EU660395 | 2007 |
| I | VN | EU660394 | 2006 |
| I | VN | EU660393 | 2006 |
| I | VN | EU660392 | 2006 |
| I | VN | EU660391 | 2006 |
| I | VN | EU660390 | 2006 |
| I | VN | EU660419 | 2007 |
| I | VN | EU660418 | 2007 |
| I | VN | EU660412 | 2007 |
| I | VN | EU660403 | 2006 |
| I | VN | EU660402 | 2006 |
| I | VN | EU660401 | 2006 |
| I | VN | EU482828 | 2006 |
| I | VN | EU482827 | 2006 |
| I | VN | EU482826 | 2006 |
| I | VN | EU482825 | 2006 |
| I | VN | EU482824 | 2006 |
| I | VN | EU482823 | 2006 |
| I | VN | EU482822 | 2006 |
| I | VN | EU482821 | 2006 |
| I | VN | EU482820 | 2006 |
| I | VN | EU482819 | 2006 |
| I | VN | EU482818 | 2006 |
| I | VN | EU482817 | 2006 |
| I | VN | EU482816 | 2006 |
| I | VN | EU482815 | 2006 |
| I | VN | EU482814 | 2006 |
| I | VN | EU482813 | 2006 |
| I | VN | EU482812 | 2006 |
| I | VN | EU482811 | 2006 |
| I | VN | EU482810 | 2006 |
| I | VN | EU482809 | 2006 |
| I | VN | EU482808 | 2006 |
| I | VN | EU482807 | 2006 |
| I | VN | EU482806 | 2006 |
| I | VN | EU482805 | 2006 |
| I | VN | EU482804 | 2006 |
| I | VN | EU482803 | 2006 |
| I | VN | EU482802 | 2006 |
| I | VN | EU482801 | 2006 |
| I | VN | EU482800 | 2006 |
| I | VN | EU482799 | 2006 |
| I | VN | EU482798 | 2006 |
| I | VN | EU482797 | 2006 |
| I | VN | EU482796 | 2006 |
| I | VN | EU482795 | 2006 |
| I | VN | EU482794 | 2006 |
| I | VN | EU482793 | 2006 |
| I | VN | EU482792 | 2003 |
| I | VN | EU482791 | 2003 |
| I | VN | EU482790 | 2003 |
| I | VN | EU482789 | 2003 |
| I | VN | EU482718 | 2007 |
| I | VN | EU482717 | 2007 |
| I | VN | EU482716 | 2007 |
| I | VN | EU482715 | 2007 |
| I | VN | EU482714 | 2007 |
| I | VN | EU482713 | 2007 |
| I | VN | EU482712 | 2007 |
| I | VN | EU482711 | 2007 |
| I | VN | EU482710 | 2007 |
| I | VN | EU482709 | 2007 |
| I | VN | EU482708 | 2007 |
| I | VN | EU482707 | 2006 |
| I | VN | EU482706 | 2006 |
| I | VN | EU482540 | 2006 |
| I | VN | EU482539 | 2006 |
| I | VN | EU482538 | 2006 |
| I | VN | EU482537 | 2006 |
| I | VN | EU482536 | 2006 |
| I | VN | EU482535 | 2006 |
| I | VN | EU482534 | 2006 |
| I | VN | EU482533 | 2006 |
| I | VN | EU482532 | 2006 |
| I | VN | EU482531 | 2006 |
| I | VN | EU482530 | 2006 |
| I | VN | EU482529 | 2006 |
| I | VN | EU482528 | 2006 |
| I | VN | EU482527 | 2006 |
| I | VN | EU482526 | 2006 |
| I | VN | EU482525 | 2006 |
| I | VN | EU482524 | 2006 |
| I | VN | EU482523 | 2006 |
| I | VN | EU482522 | 2006 |
| I | VN | EU482521 | 2006 |
| I | VN | EU482520 | 2006 |
| I | VN | EU482519 | 2006 |
| I | VN | EU482518 | 2006 |
| I | VN | EU482517 | 2007 |
| I | VN | EU482516 | 2007 |
| I | VN | EU482515 | 2007 |
| I | VN | EU482514 | 2007 |
| I | VN | EU482513 | 2007 |
| I | VN | EU482512 | 2007 |
| I | VN | EU482511 | 2007 |
| I | VN | EU482510 | 2007 |
| I | VN | EU482509 | 2007 |
| I | VN | EU482508 | 2007 |
| I | VN | EU482507 | 2007 |
| I | VN | EU482506 | 2007 |
| I | VN | EU482505 | 2007 |
| I | VN | EU482504 | 2007 |
| I | VN | EU482503 | 2007 |
| I | VN | EU482502 | 2007 |
| I | VN | EU482501 | 2007 |
| I | VN | EU482500 | 2007 |
| I | VN | EU482499 | 2007 |
| I | VN | EU482498 | 2007 |
| I | VN | EU482497 | 2007 |
| I | VN | EU482496 | 2007 |
| I | VN | EU482495 | 2007 |
| I | VN | EU482494 | 2007 |
| I | VN | EU482493 | 2007 |
| I | VN | EU482492 | 2007 |
| I | VN | EU482491 | 2007 |
| I | VN | EU482490 | 2007 |
| I | VN | EU482489 | 2007 |
| I | VN | EU482488 | 2007 |
| I | VN | EU482487 | 2007 |
| I | VN | EU482486 | 2007 |
| I | VN | EU482485 | 2007 |
| I | VN | EU482484 | 2007 |
| I | VN | EU482483 | 2007 |
| I | VN | EU482482 | 2007 |
| I | VN | EU482481 | 2007 |
| I | VN | EU482480 | 2006 |
| I | VN | EU482479 | 2007 |
| I | VN | EU482478 | 2007 |
| I | VN | EU482477 | 2007 |
| I | VN | EU482476 | 2003 |
| I | VN | EU249495 | 2006 |
| I | VN | EU249494 | 2006 |
| I | VN | EU249493 | 2006 |
| I | VN | EU249492 | 2006 |
| I | VN | EU249491 | 2006 |
| I | VN | EU249490 | 2006 |
| I | VN | FJ906728 | 2007 |
| I | VN | FJ906965 | 2007 |
| I | VN | FJ906964 | 2007 |
| I | VN | FJ906963 | 2007 |
| I | VN | FJ898431 | 2006 |
| I | VN | FJ898430 | 2006 |
| I | VN | FJ898429 | 2006 |
| I | VN | FJ898428 | 2006 |
| I | VN | FJ898427 | 2006 |
| I | VN | FJ898426 | 2006 |
| I | VN | FJ898425 | 2006 |
| I | VN | FJ898424 | 2006 |
| I | VN | FJ898423 | 2006 |
| I | VN | FJ898422 | 2006 |
| I | VN | FJ898421 | 2006 |
| I | VN | FJ898420 | 2006 |
| I | VN | FJ898419 | 2006 |
| I | VN | FJ898418 | 2006 |
| I | VN | FJ898417 | 2006 |
| I | VN | FJ898416 | 2006 |
| I | VN | FJ898415 | 2006 |
| I | VN | FJ898414 | 2006 |
| I | VN | FJ898413 | 2006 |
| I | VN | FJ898412 | 2006 |
| I | VN | FJ898411 | 2006 |
| I | VN | FJ898410 | 2006 |
| I | VN | FJ898409 | 2006 |
| I | VN | FJ898408 | 2006 |
| I | VN | FJ898407 | 2006 |
| I | VN | FJ898406 | 2006 |
| I | VN | FJ898405 | 2006 |
| I | VN | FJ898404 | 2006 |
| I | VN | FJ898403 | 2006 |
| I | VN | FJ898402 | 2006 |
| I | VN | FJ898401 | 2006 |
| I | VN | FJ898400 | 2006 |
| I | VN | FJ898399 | 2006 |
| I | VN | FJ898398 | 2006 |
| I | VN | FJ898397 | 2006 |
| I | VN | FJ898396 | 2006 |
| I | VN | FJ898395 | 2005 |
| I | VN | FJ898394 | 2005 |
| I | VN | FJ898393 | 2005 |
| I | VN | FJ898392 | 2005 |
| I | VN | FJ898391 | 2005 |
| I | VN | FJ898390 | 2005 |
| I | VN | FJ898389 | 2005 |
| I | VN | FJ898388 | 2005 |
| I | VN | FJ898387 | 2005 |
| I | VN | FJ898386 | 2005 |
| I | VN | FJ898385 | 2007 |
| I | VN | FJ898384 | 2007 |
| I | VN | FJ898383 | 2007 |
| I | VN | FJ898382 | 2007 |
| I | VN | FJ898381 | 2007 |
| I | VN | FJ898380 | 2007 |
| I | VN | FJ898379 | 2007 |
| I | VN | FJ898378 | 2007 |
| I | VN | FJ898377 | 2007 |
| I | VN | FJ898376 | 2007 |
| I | VN | FJ898375 | 2007 |
| I | VN | FJ898374 | 2007 |
| I | VN | FJ898373 | 2007 |
| I | VN | FJ898372 | 2007 |
| I | VN | FJ898371 | 2007 |
| I | VN | FJ882570 | 2005 |
| I | VN | FJ882569 | 2004 |
| I | VN | FJ882568 | 2003 |
| I | VN | FJ882567 | 2003 |
| I | VN | FJ882566 | 2003 |
| I | VN | FJ882565 | 2003 |
| I | VN | FJ882564 | 2003 |
| I | VN | FJ882563 | 2003 |
| I | VN | FJ882562 | 2003 |
| I | VN | FJ882561 | 2007 |
| I | VN | FJ882560 | 2007 |
| I | VN | FJ882559 | 2007 |
| I | VN | FJ882558 | 2007 |
| I | VN | FJ882557 | 2007 |
| I | VN | FJ882556 | 2007 |
| I | VN | FJ882555 | 2007 |
| I | VN | FJ882554 | 2007 |
| I | VN | FJ882553 | 2007 |
| I | VN | FJ882552 | 2007 |
| I | VN | FJ882551 | 2007 |
| I | VN | FJ882550 | 2007 |
| I | VN | FJ882549 | 2007 |
| I | VN | FJ882548 | 2007 |
| I | VN | FJ882547 | 2007 |
| I | VN | FJ882546 | 2007 |
| I | VN | FJ882545 | 2007 |
| I | VN | FJ882544 | 2006 |
| I | VN | FJ882543 | 2006 |
| I | VN | FJ882542 | 2006 |
| I | VN | FJ882541 | 2006 |
| I | VN | FJ882540 | 2006 |
| I | VN | FJ882539 | 2006 |
| I | VN | FJ882538 | 2006 |
| I | VN | FJ882537 | 2006 |
| I | VN | FJ882536 | 2006 |
| I | VN | FJ882535 | 2006 |
| I | VN | FJ882534 | 2006 |
| I | VN | FJ882533 | 2006 |
| I | VN | FJ882532 | 2006 |
| I | VN | FJ882531 | 2006 |
| I | VN | FJ882530 | 2006 |
| I | VN | FJ882529 | 2006 |
| I | VN | FJ882528 | 2006 |
| I | VN | FJ882527 | 2006 |
| I | VN | FJ882526 | 2006 |
| I | VN | FJ882525 | 2006 |
| I | VN | FJ882524 | 2006 |
| I | VN | FJ882523 | 2006 |
| I | VN | FJ882522 | 2006 |
| I | VN | FJ882521 | 2006 |
| I | VN | FJ882520 | 2006 |
| I | VN | FJ882519 | 2006 |
| I | VN | FJ882518 | 2006 |
| I | VN | FJ882517 | 2006 |
| I | VN | FJ882516 | 2006 |
| I | VN | FJ882515 | 2006 |
| I | VN | FJ859029 | 2007 |
| I | VN | FJ461328 | 2007 |
| I | VN | FJ461320 | 2007 |
| I | KH | FJ850069 | 2003 |
| I | TH | FJ850068 | 2001 |
| I | KH | FJ639696 | 2007 |
| I | KH | FJ639695 | 2007 |
| I | KH | FJ639694 | 2007 |
| I | KH | FJ639693 | 2007 |
| I | KH | FJ639692 | 2007 |
| I | KH | FJ639687 | 2006 |
| I | KH | FJ639683 | 2005 |
| I | KH | FJ639682 | 2004 |
| I | KH | FJ639681 | 2003 |
| I | KH | FJ639680 | 2003 |
| I | KH | FJ639679 | 2003 |
| I | KH | FJ639678 | 2003 |
| I | KH | FJ639677 | 2003 |
| I | KH | FJ639676 | 2003 |
| I | KH | FJ639675 | 2003 |
| I | KH | FJ639673 | 2001 |
| I | KH | FJ639672 | 2001 |
| I | KH | FJ639671 | 2001 |
| I | KH | FJ639670 | 2001 |
| I | KH | FJ639669 | 2000 |
| I | KH | FJ639691 | 2007 |
| I | KH | FJ639690 | 2007 |
| I | KH | FJ639689 | 2007 |
| I | KH | FJ639688 | 2007 |
| I | KH | FJ639686 | 2006 |
| I | KH | FJ639685 | 2005 |
| I | KH | FJ639684 | 2005 |
| I | KH | FJ639674 | 2002 |
| I | VN | FJ461341 | 2008 |
| I | VN | FJ461340 | 2008 |
| I | VN | FJ461339 | 2008 |
| I | VN | FJ461336 | 2008 |
| I | VN | FJ461335 | 2008 |
| I | VN | FJ461333 | 2007 |
| I | VN | FJ461332 | 2008 |
| I | VN | FJ461331 | 2008 |
| I | VN | FJ461330 | 2008 |
| I | VN | FJ461327 | 2007 |
| I | VN | FJ461325 | 2007 |
| I | VN | FJ461324 | 2007 |
| I | VN | FJ461323 | 2007 |
| I | VN | FJ461319 | 2007 |
| I | VN | FJ461318 | 2007 |
| I | VN | FJ461317 | 2007 |
| I | VN | FJ461316 | 2007 |
| I | VN | FJ461315 | 2007 |
| I | VN | FJ461313 | 2007 |
| I | VN | FJ461312 | 2007 |
| I | VN | FJ461310 | 2008 |
| I | VN | FJ461308 | 2008 |
| I | VN | FJ461307 | 2008 |
| I | VN | FJ461306 | 2007 |
| I | VN | FJ562101 | 2007 |
| I | VN | FJ547065 | 2007 |
| I | VN | FJ547063 | 2007 |
| I | VN | FJ547060 | 2007 |
| I | MY | GQ328923 | 2004 |
| I | VN | FJ461303 | 2007 |
| I | MY | FR666927 | 2005 |
| I | MY | FR666926 | 2004 |
| I | MY | FR666925 | 2005 |
| I | MY | FR666924 | 2005 |
| I | MY | FR666923 | 2004 |
| I | MY | FR666922 | 2004 |
| I | MY | FR666928 | 2005 |
| I | TH | HM134239 | 2003 |
| I | SG | GQ357689 | 2007 |
| I | SG | GQ357687 | 2008 |
| I | SG | GQ357686 | 2008 |
| I | SG | GQ357685 | 2008 |
| I | SG | GQ357684 | 2008 |
| I | SG | GQ357683 | 2008 |
| I | SG | GQ357682 | 2008 |
| I | SG | GQ357681 | 2008 |
| I | SG | GQ357680 | 2008 |
| I | SG | GQ357679 | 2008 |
| I | SG | GQ357678 | 2008 |
| I | SG | GQ357677 | 2008 |
| I | SG | GQ357676 | 2008 |
| I | SG | GQ357675 | 2008 |
| I | SG | GQ357674 | 2008 |
| I | SG | GQ357673 | 2008 |
| I | SG | GQ357672 | 2008 |
| I | SG | GQ357671 | 2008 |
| I | SG | GQ357670 | 2008 |
| I | SG | GQ357669 | 2008 |
| I | SG | GQ357668 | 2008 |
| I | SG | GQ357667 | 2008 |
| I | SG | GQ357666 | 2008 |
| I | MY(KR) | FJ687478 | 2008 |
| I | ID(KR) | FJ687477 | 2008 |
| I | TH(KH) | FJ687474 | 2007 |
| I | ID | EU448401 | 2007 |
| I | MY | EU448400 | 2006 |
| I | SG | EU448399 | 2005 |
| I | TH | EU448398 | 2007 |
| I | VN | EU448397 | 2007 |
| I | TH | EU448396 | 2003 |
| I | MY | EU448395 | 2007 |
| I | TH | EU448394 | 2007 |
| I | TH | EU448393 | 2003 |
| I | VN | EU448392 | 2007 |
| I | ID | EU448391 | 2006 |
| I | VN | EU448390 | 2007 |
| I | KH | EU448389 | 2007 |
| I | VN | EU448388 | 2007 |
| I | VN | EU448387 | 2004 |
| I | TH | EU448386 | 2004 |
| I | TH | EU117312 | 2001 |
| I | TH | EU117311 | 2001 |
| I | TH | EU117310 | 2001 |
| I | TH | EU117309 | 2001 |
| I | TH | EU117308 | 2001 |
| I | TH | EU117307 | 2001 |
| I | TH | EU117306 | 2001 |
| I | TH | EU117305 | 2001 |
| I | TH | EU117304 | 2001 |
| I | SG | EU069624 | 2004 |
| I | SG | EU069623 | 2004 |
| I | SG | EU069622 | 2004 |
| I | SG | EU069621 | 2004 |
| I | SG | EU069620 | 2004 |
| I | SG | EU069619 | 2004 |
| I | SG | EU069618 | 2004 |
| I | SG | EU069617 | 2003 |
| I | SG | EU069616 | 2003 |
| I | SG | EU069615 | 2003 |
| I | SG | EU069614 | 2003 |
| I | SG | EU069613 | 2004 |
| I | SG | EU069612 | 2003 |
| I | SG | EU069610 | 2003 |
| I | SG | EU069609 | 2004 |
| I | SG | EU069608 | 2003 |
| I | SG | EU069607 | 2003 |
| I | SG | EU069606 | 2004 |
| I | SG | EU069605 | 2004 |
| I | SG | EU069604 | 2005 |
| I | SG | EU069603 | 2004 |
| I | SG | EU069602 | 2005 |
| I | SG | EU069601 | 2003 |
| I | SG | EU069600 | 2002 |
| I | SG | EU069599 | 2002 |
| I | SG | EU069598 | 2003 |
| I | SG | EU069596 | 2005 |
| I | SG | EU069595 | 2005 |
| I | SG | EU069594 | 2005 |
| I | SG | EU069593 | 2004 |
| I | CN | EF508207 | 2006 |
| I | CN | EF508206 | 2006 |
| I | CN | EF508205 | 2006 |
| I | CN | EF508204 | 2006 |
| I | CN | EF508203 | 2006 |
| I | CN | EF508202 | 2004 |
| I | CN | EF508200 | 2001 |
| I | CN | EF508199 | 1999 |
| I | CN | EF508198 | 1998 |
| I | MM | DQ264984 | 2002 |
| I | MM | DQ264983 | 2002 |
| I | MM | DQ264982 | 2002 |
| I | MM | DQ264981 | 2002 |
| I | MM | DQ264980 | 2002 |
| I | MM | DQ264979 | 2002 |
| I | MM | DQ264974 | 2002 |
| I | MM | DQ264973 | 2002 |
| I | MM | DQ264971 | 2002 |
| I | MM | DQ264970 | 2002 |
| I | TH | AY732473 | 1995 |
| I | TH | AY732472 | 1998 |
| I | TH | AY732471 | 1992 |
| I | TH | AY732470 | 1999 |
| I | TH | AY732469 | 2000 |
| I | TH | AY732468 | 1994 |
| I | TH | AY732467 | 2001 |
| I | TH | AY732466 | 1990 |
| I | TH | AY732465 | 1993 |
| I | TH | AY732464 | 2001 |
| I | TH | AY732463 | 1993 |
| I | TH | AY732462 | 2001 |
| I | TH | AY732461 | 1993 |
| I | TH | AY732460 | 2000 |
| I | TH | AY732459 | 1997 |
| I | TH | AY732458 | 1999 |
| I | TH | AY732457 | 1995 |
| I | TH | AY732456 | 1994 |
| I | TH | AY732455 | 1996 |
| I | TH | AY732454 | 1998 |
| I | TH | AY732453 | 1997 |
| I | TH | AY732452 | 2000 |
| I | TH | AY732451 | 2001 |
| I | TH | AY732450 | 1997 |
| I | TH | AY732449 | 1999 |
| I | TH | AY732448 | 1990 |
| I | TH | AY732446 | 1999 |
| I | TH | AY732445 | 1987 |
| I | TH | AY732444 | 1997 |
| I | TH | AY732443 | 1999 |
| I | TH | AY732442 | 1990 |
| I | TH | AY732441 | 1990 |
| I | TH | AY732440 | 1987 |
| I | TH | AY732439 | 1999 |
| I | TH | AY732438 | 2001 |
| I | TH | AY732437 | 1988 |
| I | TH | AY732436 | 1991 |
| I | TH | AY732435 | 1987 |
| I | TH | AY732434 | 1998 |
| I | TH | AY732433 | 1989 |
| I | TH | AY732432 | 1986 |
| I | TH | AY732431 | 1994 |
| I | TH | AY732430 | 1995 |
| I | TH | AY732428 | 2001 |
| I | TH | AY732427 | 1994 |
| I | TH | AY732426 | 1985 |
| I | TH | AY732425 | 1987 |
| I | TH | AY732424 | 1993 |
| I | TH | AY732423 | 1996 |
| I | TH | AY732422 | 1996 |
| I | TH | AY732421 | 1980 |
| I | TH | AY732420 | 1986 |
| I | TH | AY732419 | 2001 |
| I | TH | AY732418 | 1997 |
| I | TH | AY732417 | 1997 |
| I | TH | AY732416 | 1997 |
| I | TH | AY732415 | 1993 |
| I | TH | AY732414 | 1991 |
| I | TH | AY732413 | 1991 |
| I | TH | AY732412 | 1992 |
| I | TH | AY732410 | 1989 |
| I | TH | AY732409 | 2000 |
| I | TH | AY732408 | 2000 |
| I | TH | AY732407 | 2000 |
| I | TH | AY732406 | 1981 |
| I | TH | AY732405 | 1992 |
| I | TH | AY732404 | 1995 |
| I | TH | AY732403 | 2002 |
| I | TH | AY732402 | 1992 |
| I | TH | AY732401 | 2001 |
| I | TH | AY732400 | 1999 |
| I | TH | AY732399 | 1993 |
| I | TH | AY732398 | 2002 |
| I | TH | AY732397 | 1982 |
| I | TH | AY732396 | 1998 |
| I | TH | AY732395 | 1992 |
| I | TH | AY732394 | 1981 |
| I | TH | AY732393 | 1994 |
| I | TH | AY732392 | 2001 |
| I | TH | AY732391 | 1997 |
| I | TH | AY732390 | 1983 |
| I | TH | AY732389 | 2001 |
| I | TH | AY732388 | 1989 |
| I | TH | AY732387 | 1995 |
| I | TH | AY732386 | 2002 |
| I | TH | AY732385 | 1981 |
| I | TH | AY732384 | 1988 |
| I | TH | AY732383 | 1981 |
| I | TH | AY732382 | 1991 |
| I | TH | AY732381 | 1988 |
| I | TH | AY732380 | 1988 |
| I | TH | AF425630 | 1980 |
| I | TW | AF425628 | 1987 |
| I | TH(KR) | EF654109 | 2005 |
| I | TH(KR) | EF654108 | 2005 |
| I | ID(KR) | EF654107 | 2005 |
| I | ID(KR) | EF654106 | 2005 |
| I | ID(KR) | EF654105 | 2005 |
| I | CN | EF113152 | 2006 |
| I | CN | AY871812 | 2004 |
| I | MM | AY618880 | 2000 |
| I | MM | AY618879 | 2000 |
| I | MM | AY618878 | 1999 |
| I | MM | AY618877 | 2000 |
| I | MM | AY620953 | 2001 |
| I | MM | AY620952 | 2000 |
| I | MM | AY620951 | 2000 |
| I | MM | AY620950 | 1999 |
| I | MM | AY620949 | 2001 |
| I | MM | AY620948 | 2001 |
| I | MM | AY620947 | 2001 |
| I | MM | AY620946 | 2001 |
| I | MM | AY618211 | 2001 |
| I | MM | AY618210 | 2001 |
| I | MM | AY606062 | 2001 |
| I | MM | AY588273 | 1998 |
| I | MM | AY588272 | 1998 |
| I | TH(JP) | AB111079 | 2002 |
| I | TH(JP) | AB111078 | 2002 |
| I | TH(JP) | AB111077 | 2002 |
| I | TH(JP) | AB111076 | 2002 |
| I | TH(JP) | AB111072 | 2001 |
| I | KH(JP) | AB111071 | 2001 |
| I | KH(JP) | AB111069 | 2001 |
| I | SG MY TH (JP) | AB111067 | 2001 |
| I | IN TH (JP) | AB111066 | 2001 |
| I | TH (JP) | AB111064 | 1998 |
| I | CN | FJ158612 | 2007 |
| I | CN | FJ158611 | 2007 |
| I | CN | FJ158610 | 2007 |
| I | CN | FJ158609 | 2007 |
| I | SA | AM746217 | 2004 |
| I | SA | AM746216 | 2004 |
| I | SA | AM746215 | 2005 |
| I | SA | AM746214 | 2005 |
| I | SA | AM746213 | 2006 |
| I | SA | AM746212 | 2006 |
| I | LA | AB003090 | 1996 |
| IV | AW | D00505 | 1985 |
| IV | HI(US) | DQ672564 | 2001 |
| IV | HI(US) | DQ672563 | 2001 |
| IV | HI(US) | DQ672562 | 2001 |
| IV | HI(US) | DQ672561 | 2001 |
| IV | HI(US) | DQ672560 | 2001 |
| IV | PH | GQ868602 | 2004 |
| IV | CN | EF025110 | 1971 |
| IV | PF | FJ898448 | 2001 |
| IV | BN | EU179861 | 2006 |
| IV | ID | AB189121 | 1998 |
| IV | ID | AB189120 | 1998 |
| IV | JP | AB204803 | 2004 |
| IV | SC | AB195673 | 2003 |
| IV | CL | EU863650 | 2002 |
| IV | CN | EF032590 | 1995 |
| IV | MY | FR666920 | 2004 |
| IV | MY | FR666921 | 2004 |
| IV | KR(PH) | FJ687476 | 2007 |
| IV | PH | AY422777 | 2001 |
| IV | MG | EU448412 | 2006 |
| IV | ID | EU448411 | 2005 |
| IV | MY | EU448410 | 2004 |
| IV | ID | EU448409 | 2007 |
| IV | ID | EU448408 | 2003 |
| IV | ID | EU448407 | 2005 |
| IV | PH | EU448406 | 2003 |
| IV | PH | EU448405 | 2007 |
| IV | ID | EU448404 | 2003 |
| IV | VN | EU448403 | 2006 |
| IV | ID | EU448402 | 2006 |
| IV | SG | EU069611 | 2003 |
| IV | SG | EU069597 | 2005 |
| IV | CN | EF508201 | 2002 |
| IV | SC | DQ285557 | 2004 |
| IV | RE | DQ285553 | 2004 |
| IV | RE | DQ285552 | 2004 |
| IV | RE | DQ285551 | 2004 |
| IV | RE | DQ285549 | 2004 |
| IV | HI(US) | DQ091273 | 2001 |
| IV | HI(US) | DQ091272 | 2001 |
| IV | HI(US) | DQ091271 | 2001 |
| IV | HI(US) | DQ091270 | 2001 |
| IV | HI(US) | DQ091269 | 2001 |
| IV | HI(US) | DQ091268 | 2001 |
| IV | HI(US) | DQ091267 | 2001 |
| IV | HI(US) | DQ091266 | 2001 |
| IV | HI(US) | DQ091265 | 2001 |
| IV | HI(US) | DQ091264 | 2001 |
| IV | HI(US) | DQ091263 | 2001 |
| IV | HI(US) | DQ091262 | 2001 |
| IV | HI(US) | DQ091261 | 2001 |
| IV | HI(US) | DQ091260 | 2001 |
| IV | HI(US) | DQ091259 | 2001 |
| IV | HI(US) | DQ091258 | 2001 |
| IV | PH | AF425627 | 1974 |
| IV | AU | AF425612 | 1983 |
| IV | AU | AF425611 | 1983 |
| IV | PH(KR) | EF654110 | 2006 |
| IV | CN | EF079826 | 2002 |
| IV | CN | DQ855297 | 1995 |
| IV | CN | DQ855296 | 2002 |
| IV | CN | DQ211349 | 1993 |
| IV | CN | DQ211348 | 1993 |
| IV | PH | AY422786 | 1999 |
| IV | PH | AY422785 | 1999 |
| IV | PH | AY422783 | 2002 |
| IV | PH | AY422782 | 2002 |
| IV | PH | AY422781 | 2002 |
| IV | PH | AY422780 | 2002 |
| IV | PH | AY422779 | 2002 |
| IV | PH | AY422778 | 2002 |
| IV | PF | AY630407 | 2001 |
| IV | ID(JP) | AB111075 | 2002 |
| IV | PH(JP) | AB111074 | 2002 |
| IV | ID(JP) | AB111073 | 2002 |
| IV | PF(JP) | AB111070 | 2001 |
| IV | WS(JP) | AB111068 | 2001 |
| IV | ID | AB232666 | 2002 |
| IV | RE | EU282328 | 2004 |
| IV | MX | D00504 | 1983 |
| V | TH | AY732379 | 1983 |
| V | IN(KR) | FJ687475 | 2006 |
| V | MX | HQ166037 | 2008 |
| V | MX | HQ166036 | 2007 |
| V | MX | HQ166035 | 2007 |
| V | VE | FJ744701 | 2004 |
| V | MX | HM631855 | 2007 |
| V | MX | GU131984 | 2008 |
| V | MX | GU131983 | 2008 |
| V | MX | GU131982 | 2008 |
| V | MX | GU131981 | 2007 |
| V | MX | GU131980 | 2007 |
| V | MX | GU131979 | 2007 |
| V | MX | GU131978 | 2007 |
| V | MX | GU131977 | 2007 |
| V | MX | GU131976 | 2007 |
| V | MX | GU131973 | 2007 |
| V | MX | GU131972 | 2007 |
| V | MX | GU131971 | 2007 |
| V | MX | GU131970 | 2007 |
| V | MX | GU131969 | 2007 |
| V | MX | GU131968 | 2007 |
| V | MX | GU131967 | 2007 |
| V | MX | GU131966 | 2007 |
| V | MX | GU131965 | 2007 |
| V | MX | GU131964 | 2007 |
| V | MX | GU131963 | 2007 |
| V | MX | GU131962 | 2007 |
| V | MX | GU131961 | 2007 |
| V | MX | GU131960 | 2007 |
| V | MX | GU131958 | 2006 |
| V | MX | GU131957 | 2006 |
| V | MX | GU131956 | 2006 |
| V | CO | GU131949 | 2006 |
| V | CO | GU131948 | 2001 |
| V | CI | AF298807 | 1998 |
| V | BR | AF226685 | 1990 |
| V | BR | AF513110 | 2001 |
| V | GF | AF226687 | 1989 |
| V | SG | EU081258 | 2005 |
| V | TH | AY732476 | 1980 |
| V | TH | AY732474 | 1980 |
| V | PY(AR) | AY277666 | 2000 |
| V | AR | AY277665 | 2000 |
| V | AR | AY277664 | 1999 |
| V | AR | AY277659 | 2000 |
| V | AR | AY277654 | 2000 |
| V | BR | GU131863 | 2008 |
| V | VE | GU131842 | 2007 |
| V | VE | GU131841 | 2007 |
| V | VE | GU131840 | 2007 |
| V | VE | GU131839 | 2006 |
| V | VE | GU131838 | 2006 |
| V | VE | GU131837 | 2005 |
| V | VE | GU131836 | 2004 |
| V | VE | GU131835 | 2004 |
| V | VE | GU131834 | 2001 |
| V | VE | GU131833 | 2000 |
| V | VE | GU131832 | 2000 |
| V | VE | GU056033 | 1998 |
| V | VE | GU056032 | 1998 |
| V | VE | GU056031 | 1998 |
| V | VE | GU056030 | 1997 |
| V | VE | GU056029 | 1997 |
| V | MX | GQ868539 | 2008 |
| V | CO | GQ868570 | 2008 |
| V | CO | GQ868569 | 2007 |
| V | CO | GQ868568 | 2007 |
| V | CO | GQ868567 | 2007 |
| V | CO | GQ868566 | 2007 |
| V | CO | GQ868565 | 2006 |
| V | CO | GQ868564 | 2006 |
| V | CO | GQ868563 | 2006 |
| V | CO | GQ868562 | 2005 |
| V | CO | GQ868561 | 1999 |
| V | CO | GQ868560 | 1998 |
| V | CO | GQ868559 | 1998 |
| V | MX | GQ868538 | 2008 |
| V | MX | GQ868537 | 2008 |
| V | MX | GQ868536 | 2008 |
| V | MX | GQ868535 | 2008 |
| V | MX | GQ868534 | 2008 |
| V | MX | GQ868533 | 2008 |
| V | MX | GQ868532 | 2008 |
| V | MX | GQ868531 | 2008 |
| V | MX | GQ868530 | 2008 |
| V | MX | GQ868529 | 2008 |
| V | MX | GQ868528 | 2007 |
| V | MX | GQ868527 | 2007 |
| V | MX | GQ868526 | 2007 |
| V | MX | GQ868525 | 2007 |
| V | MX | GQ868524 | 2007 |
| V | MX | GQ868523 | 2007 |
| V | MX | GQ868522 | 2007 |
| V | MX | GQ868521 | 2007 |
| V | MX | GQ868520 | 2007 |
| V | MX | GQ868519 | 2007 |
| V | MX | GQ868518 | 2007 |
| V | MX | GQ868517 | 2007 |
| V | MX | GQ868514 | 2007 |
| V | MX | GQ868513 | 2007 |
| V | MX | GQ868512 | 2007 |
| V | MX | GQ868511 | 2007 |
| V | MX | GQ868510 | 2007 |
| V | MX | GQ868509 | 2007 |
| V | MX | GQ868508 | 2007 |
| V | MX | GQ868507 | 2007 |
| V | MX | GQ868506 | 2007 |
| V | MX | GQ868505 | 2007 |
| V | MX | GQ868504 | 2007 |
| V | MX | GQ868503 | 2007 |
| V | MX | GQ868502 | 2007 |
| V | MX | GQ868501 | 2007 |
| V | MX | GQ868500 | 2007 |
| V | MX | GQ868499 | 2006 |
| V | MX | GQ868498 | 2006 |
| V | VG | GQ868601 | 1985 |
| V | VE | GQ199877 | 2007 |
| V | NI | GQ199875 | 2004 |
| V | NI | GQ199873 | 2004 |
| V | NI | GQ199872 | 2004 |
| V | NI | GQ199867 | 2004 |
| V | NI | GQ199859 | 2008 |
| V | NI | GQ199858 | 2008 |
| V | NI | GQ199857 | 2008 |
| V | NI | FJ898437 | 2004 |
| V | NI | FJ898433 | 2007 |
| V | NI | FJ873814 | 2005 |
| V | NI | FJ547089 | 2005 |
| V | NI | FJ432720 | 2005 |
| V | NI | FJ410290 | 2005 |
| V | NI | FJ182002 | 2005 |
| V | NI | FJ024485 | 2005 |
| V | NI | FJ024484 | 2005 |
| V | NI | FJ024483 | 2005 |
| V | NI | FJ024482 | 2005 |
| V | NI | FJ024481 | 2005 |
| V | NI | FJ024480 | 2005 |
| V | NI | FJ024479 | 2006 |
| V | NI | FJ024478 | 2005 |
| V | NI | FJ024423 | 2005 |
| V | NI | EU596504 | 2005 |
| V | NI | EU596503 | 2005 |
| V | NI | EU596502 | 2005 |
| V | NI | EU596501 | 2004 |
| V | SG | M87512 | 1990 |
| V | MM | AY722803 | 1998 |
| V | MM | AY722802 | 1996 |
| V | MM | AY722801 | 1976 |
| V | MM | AY713473 | 1971 |
| V | SG | AY762084 | 1993 |
| V | BR | AF311958 | 1997 |
| V | BR | AF311957 | 1997 |
| V | BR | AF311956 | 1997 |
| V | BR | AB519681 | 2001 |
| V | NI | FJ850114 | 2005 |
| V | NI | FJ850113 | 2005 |
| V | NI | FJ547088 | 2008 |
| V | NI | FJ432721 | 2005 |
| V | US | FJ410190 | 1987 |
| V | US | FJ410189 | 1996 |
| V | US | FJ410188 | 1996 |
| V | US | FJ410187 | 1992 |
| V | US | FJ410186 | 1992 |
| V | US | FJ410185 | 1993 |
| V | US | FJ410184 | 1993 |
| V | US | FJ410183 | 1993 |
| V | US | FJ410182 | 1996 |
| V | US | FJ410181 | 1995 |
| V | US | FJ410180 | 1995 |
| V | US | FJ410179 | 1994 |
| V | US | FJ410175 | 1994 |
| V | US | FJ410174 | 1995 |
| V | US | FJ410173 | 1998 |
| V | US | FJ390380 | 1998 |
| V | US | FJ390379 | 1998 |
| V | US | FJ390378 | 1998 |
| V | US | FJ390374 | 1995 |
| V | US | FJ205875 | 1995 |
| V | US | FJ205874 | 1995 |
| V | US | FJ205873 | 1998 |
| V | US | FJ205872 | 1998 |
| V | NI | EU482619 | 2005 |
| V | NI | EU482618 | 2005 |
| V | NI | EU482617 | 2005 |
| V | NI | EU482616 | 2005 |
| V | NI | EU482615 | 2005 |
| V | VE | EU482611 | 2007 |
| V | VE | EU482610 | 2007 |
| V | VE | EU482609 | 2007 |
| V | US | EU482592 | 1998 |
| V | US | EU482591 | 2006 |
| V | US | EU482567 | 1998 |
| V | VE | FJ882579 | 2007 |
| V | VE | FJ873810 | 2007 |
| V | VE | FJ873809 | 2007 |
| V | VE | FJ850104 | 2008 |
| V | VE | FJ850103 | 2008 |
| V | VE | FJ850102 | 2007 |
| V | VE | FJ850101 | 2007 |
| V | VE | FJ850100 | 2007 |
| V | VE | FJ850099 | 2007 |
| V | BR | FJ850093 | 2008 |
| V | BR | FJ850090 | 2007 |
| V | BR | FJ850087 | 2006 |
| V | BR | FJ850084 | 2005 |
| V | BR | FJ850081 | 2004 |
| V | BR | FJ850077 | 2003 |
| V | BR | FJ850075 | 2002 |
| V | BR | FJ850073 | 2001 |
| V | BR | FJ850071 | 2000 |
| V | BR | FJ850070 | 2000 |
| V | NI | FJ810419 | 2006 |
| V | VE | FJ810415 | 2005 |
| V | VE | FJ639824 | 2006 |
| V | VE | FJ639823 | 2006 |
| V | VE | FJ639821 | 2006 |
| V | VE | FJ639820 | 2006 |
| V | VE | FJ639819 | 2006 |
| V | VE | FJ639818 | 2006 |
| V | VE | FJ639815 | 2006 |
| V | VE | FJ639814 | 2005 |
| V | VE | FJ639813 | 2005 |
| V | VE | FJ639812 | 2005 |
| V | VE | FJ639811 | 2005 |
| V | VE | FJ639808 | 2005 |
| V | VE | FJ639806 | 2007 |
| V | VE | FJ639802 | 2004 |
| V | VE | FJ639797 | 2004 |
| V | VE | FJ639796 | 2004 |
| V | VE | FJ639794 | 2004 |
| V | VE | FJ639743 | 1999 |
| V | VE | FJ639741 | 1998 |
| V | VE | FJ639740 | 1998 |
| V | VE | FJ639735 | 1997 |
| V | NI | FJ547068 | 2006 |
| V | US | FJ562106 | 1986 |
| V | US | FJ562105 | 1993 |
| V | NI | FJ562104 | 2006 |
| V | US | FJ547087 | 1992 |
| V | US | FJ547086 | 1995 |
| V | US | FJ478458 | 1987 |
| V | US | FJ478457 | 1996 |
| V | MX | HM171570 | 2006 |
| V | MX | HM171569 | 2007 |
| V | MX | HM171568 | 2006 |
| V | MX | HM171567 | 2006 |
| V | MX | HM171566 | 2006 |
| V | MX | HM171565 | 2007 |
| V | MX | HM171564 | 2007 |
| V | MX | HM171563 | 2007 |
| V | MX | HM171562 | 2006 |
| V | MX | HM171561 | 2007 |
| V | MX | HM171560 | 2006 |
| V | MX | HM171559 | 2006 |
| V | MX | HM171558 | 2006 |
| V | MX | HM171557 | 2006 |
| V | PR | AY780642 | 1994 |
| V | SG | GQ357692 | 2008 |
| V | SG | GQ357691 | 2007 |
| V | SG | GQ357690 | 2007 |
| V | SV | EU448414 | 2006 |
| V | IN | EU448413 | 2006 |
| V | MX | DQ341194 | 1995 |
| V | MX | DQ341193 | 1995 |
| V | MX | DQ341192 | 1994 |
| V | MX | DQ341191 | 1986 |
| V | MX | DQ341190 | 1984 |
| V | MX | DQ341189 | 1984 |
| V | MX | DQ341188 | 1982 |
| V | RE | DQ285554 | 2004 |
| V | TH | AY732447 | 1980 |
| V | TH | AY732429 | 1980 |
| V | TH | AY732411 | 1980 |
| V | TT | AF425639 | 1986 |
| V | VE | AF425638 | 1995 |
| V | VE | AF425637 | 1994 |
| V | VE | AF425636 | 1994 |
| V | VE | AF425635 | 1995 |
| V | VE | AF425634 | 1997 |
| V | VE | AF425633 | 1995 |
| V | VE | AF425632 | 1995 |
| V | TT | AF425631 | 1978 |
| V | PE | AF425626 | 1991 |
| V | NG | AF425625 | 1968 |
| V | MX | AF425624 | 1983 |
| V | MX | AF425623 | 1980 |
| V | JM | AF425621 | 1977 |
| V | CI | AF425620 | 1985 |
| V | GD | AF425618 | 1977 |
| V | CO | AF425617 | 1996 |
| V | CO | AF425616 | 1985 |
| V | MM | AF425615 | 1976 |
| V | BR | AF425614 | 1997 |
| V | BR | AF425613 | 1982 |
| V | AO | AF425610 | 1988 |
| V | AW | AF425609 | 1985 |
| V | PF | AY630408 | 1989 |
| V | IN SG (KR) | EF654104 | 2004 |
| V | MM | AY600860 | 1998 |
| V | MM | AY589692 | 1996 |
| V | CR | AY153755 | 1993 |
| V | PY (JP) | AB111065 | 1999 |
| V | SA | AM746220 | 1994 |
| V | SA | AM746219 | 1994 |
| V | SA | AM746218 | 1994 |
| III | MY | EF457905 | 1972 |
| III | MY | FN825674 | 2005 |
| III | MY | AF425622 | 1972 |
| III | MY | AF231721 | 1972 |
