# Supplementary material for: Worldwide Spread of Dengue Virus Type 1
Source: PLoS One. 2013 May 13;8(5):e62649. doi: 10.1371/journal.pone.0062649 (PMC3652851; doi:10.1371/journal.pone.0062649)
Supplement: Table S2 — The significant connections at Bayes factor 20 to establish epidemiological linkage in viral phylogeographic histories. (DOCX) [file pone.0062649.s003.docx]

**Table S2.** The significant connections at Bayes factor 20 to establish epidemiological linkage in viral phylogeographic histories.

| **Dataset** | **BF** | **Between** |
| --- | --- | --- |
| **Genotype I** | 101588.29 | KH and VN |
|  | 639.99 | SG and MY |
|  | 474.83 | TH and CN |
|  | 85.93 | MM and TH |
|  | 85.66 | SG and ID |
|  | 69.10 | VN and ID |
|  | 26.19 | CN and KH |
|  | 23.00 | MY and CN |
|  | 20.15 | TH and SG |
| **Genotype IV** | 50.47 | SC and RE |
|  | 30.42 | RE and MG |
|  | 27.8 | WS and US (Hawaii) |
|  | 23.21 | PH and WS |
| **Genotype V** | 182151.97 | NI and MX |
|  | 2196.37 | VE and CO |
|  | 81.67 | NI and SV |
|  | 49.89 | BR and PY |
|  | 48.52 | PR and PY |
|  | 41.04 | IN and SG |
|  | 37.98 | CO and BI |
|  | 34.98 | CR and PR |
|  | 30.26 | SG and IN |
|  | 29.74 | NG and CI |
|  | 26.3 | VE and BR |
