# Supplementary material for: Worldwide Spread of Dengue Virus Type 1
Source: PLoS One. 2013 May 13;8(5):e62649. doi: 10.1371/journal.pone.0062649 (PMC3652851; doi:10.1371/journal.pone.0062649)
Supplement: Table S3 — Estimates of Net Evolutionary Divergence between groups of sequences for the complete DENV-1 dataset. Analyses were conducted using the Maximum Composite Likelihood model. The rate variation among sites was modeled with a gamma distribution (shape parameter = 0.294). (DOCX) [file pone.0062649.s004.docx]

**Table S3.** Estimates of Net Evolutionary Divergence between groups of sequences for the complete DENV-1 dataset. Analyses were conducted using the Maximum Composite Likelihood model. The rate variation among sites was modeled with a gamma distribution (shape parameter = 0.294).

|  | **Hawaiian 1944/1945** | **Genotype I** | **Genotype II** | **Genotype III** | **Genotype IV** | **Genotype V** |
| --- | --- | --- | --- | --- | --- | --- |
| **Hawaiian 1944/1945** | - |  |  |  |  |  |
| **Genotype I** | 0.057 | - |  |  |  |  |
| **Genotype II** | 0.064 | 0.093 | - |  |  |  |
| **Genotype III** | 0.068 | 0.098 | 0.067 | - |  |  |
| **Genotype IV** | 0.069 | 0.097 | 0.072 | 0.077 | - |  |
| **Genotype V** | 0.075 | 0.104 | 0.084 | 0.082 | 0.096 | - |
